# Functionalized Hybridization of 2D Nanomaterials

**DOI:** 10.1002/advs.201901837

**Published:** 2019-10-14

**Authors:** Guijian Guan, Ming‐Yong Han

**Affiliations:** ^1^ Institute of Molecular Plus Tianjin University Tianjin 300072 P. R. China; ^2^ Institute of Materials Research and Engineering A*STAR 2 Fusionopolis Way Singapore 138634 Singapore

**Keywords:** 2D nanomaterials, functionalization, heterostructures, hybrid, modification

## Abstract

The discovery of graphene and subsequent verification of its unique properties have aroused great research interest to exploit diversified graphene‐analogous 2D nanomaterials with fascinating physicochemical properties. Through either physical or chemical doping, linkage, adsorption, and hybridization with other functional species into or onto them, more novel/improved properties are readily created to extend/expand their functionalities and further achieve great performance. Here, various functionalized hybridizations by using different types of 2D nanomaterials are overviewed systematically with emphasis on their interaction formats (e.g., in‐plane or inter plane), synergistic properties, and enhanced applications. As the most intensely investigated 2D materials in the post‐graphene era, transition metal dichalcogenide nanosheets are comprehensively investigated through their element doping, physical/chemical functionalization, and nanohybridization. Meanwhile, representative hybrids with more types of nanosheets are also presented to understand their unique surface structures and address the special requirements for better applications. More excitingly, the van der Waals heterostructures of diverse 2D materials are specifically summarized to add more functionality or flexibility into 2D material systems. Finally, the current research status and faced challenges are discussed properly and several perspectives are elaborately given to accelerate the rational fabrication of varied and talented 2D hybrids.

## Graphical abstract

Through physical or chemical hybridization with other functional species into or onto 2D nanomaterials, more novel/improved properties are readily created to extend/expand their functionalities and further achieve great performances. Here, various functionalized hybridizations using different types of 2D nanomaterials are overviewed systematically with emphasis on their interaction formats (e.g., in‐plane or inter‐plane), synergistic properties, and enhanced applications.

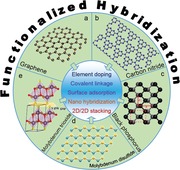

## 1 Introduction

2D nanomaterials with a large lateral size and extremely small thickness (i.e., nanosheets) have been attracting considerable efforts arising from their fascinating physicochemical properties and potential applications in energy, catalysis, detection, electronics, and optoelectronics.^1^, ^2^, ^3^, ^4^, ^5^, ^6^, ^7^, ^8^, ^9^ Mono‐ and few‐layer nanosheets with covalently bonded lattice (in‐plane) are easily fabricated by physical and chemical exfoliation of van der Waals layered counterparts (e.g., graphite to graphene).^2^ In addition to their intriguing electronic, optical and optoelectronic properties, the excellent mechanical strength and flexibility of nanosheets (Young's modulus: 1 TPa for graphene, 270 GPa for MoS_2_ monolayer compared to 240 GPa for bulk MoS_2_ and 205 GPa for steel.^8^, ^9^) have aroused more interest to develop diversified types of 2D nanosheets with improved properties and functionalities for better applications.^4^, ^5^, ^6^ Currently, there is an emphasized investigation of 2D nanosheets on their exterior morphological control in size and shape, the corresponding interior compositional and structural control of individual layers are also critical to contribute in understanding fundamental properties and technological importance.^10^, ^11^, ^12^, ^13^, ^14^

**Figure 1:**
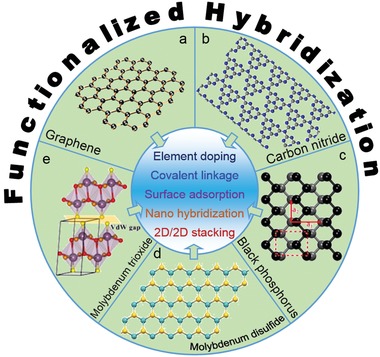
Functionalized hybridization of various 2D nanomaterials via diverse strategies to combine different species.

In the last decades, diversified 2D nanosheets with different atomic structures and compositions have been extensively investigated via their systematic fabrication, in‐depth characterization, and novel applications. For example, atomically thin graphene with a hexagonal close‐packed network is formed via covalent bonding (σ bonds) among neighboring carbons (**Figure** **1**a).^15^ Similarly, atomically thin graphitic carbon nitride with large periodic vacancies in lattice is also formed by condensing tri‐s‐triazine subunits through sp^2^ hybridization of carbon and nitrogen atoms via connecting planar tertiary amino groups (Figure 1b).^16^ Instead of the single layer of atoms, two layers of atoms are demonstrated in black phosphorus monolayer comprised of sp^3^‐hybridised phosphorus atoms with tetrahedral bonding, resulting in a puckered honeycomb structure containing armchair and zig‐zag directions of phosphorene (Figure 1c).^17^ Meanwhile, three layers of atoms are displayed in a transition metal dichalcogenide (TMD) monolayer by sandwiching a transition metal layer between two chalcogen layers (Figure 1d shows the top view of MoS_2_ monolayer).^18^ Besides TMDs, van der Waals layered MoO_3_ is also exfoliated to obtain its monolayer with four atomic layers by sharing edges of adjacent MoO_6_ octahedra in an orthorhombic crystal (Figure 1e).^19^

More interestingly, there has been a growing interest to incorporate specific species into/onto 2D nanosheets to create/endow various hybridized nanostructures with new/improved properties and synergistic functionalities for achieving excellent performance in advanced applications. Typically, elemental doping into in‐plane lattice is very useful to alter their band structure and tune their electronic, optical, and magnetic features^20^, ^21^ while surface functionalization (e.g., covalent linkage and physical adsorption) is readily to improve their stability, processability, and biocompatibility.^10^, ^20^, ^22^, ^23^, ^24^ Together with various integrations of 2D nanosheets with other nanostructures (0D or 1D),^20^, ^25^, ^26^, ^27^, ^28^, ^29^ diversified hybridized 2D nanomaterials are also facilely fabricated by combining the same/different types of 2D nanosheets, which offer new strategies for band‐structure engineering through simultaneous control of individual layers and their interfaces in the multilayered heterostructures to reduce the amount of charge displacement within each layers and increase the charge transfer between adjacent layers.^30^, ^31^, ^32^

Since the first review on 2D nanomaterials in 2001,^33^ 368 reviews have been published so far in this field, comprising of 325 reviews in the past five years to indicate the fast expansion in exploration and exploitation of diversified 2D nanomaterials. Among them, a number of reviews emphasize more on the progressive advances in the hybridized 2D nanomaterials.^23^, ^26^, ^27^, ^28^, ^29^, ^34^, ^35^ For example, one of the pioneered groups contributed the earlier four reviews with more focus on epitaxial growth of metal, metal oxide and other nanostructures on TMD and graphene nanosheets,^34^, ^35^ while a few other groups contributed reviews with more emphasis on specific applications of different hybrids in energy storage, plasmonics, and electrochemical sensing.^23^, ^26^, ^27^, ^28^ With more and more reports on the hybridized 2D nanomaterials in recent years,^29^ it is time to review the rapid progress on the hybridization of various functional nanostructures on diversified 2D nanomaterials, especially the hybridization between/among 2D nanosheets that are not collectively reviewed previously. It will be the first review to cover most of representative hybridized 2D nanomaterials.

In the review, the recent developments and important achievements on functionalized hybridization of various 2D nanomaterials are overviewed comprehensively to provide readers with a timely update and systematical knowledge for the state‐of‐the‐art progress in this dynamic research field (Figure 1). To understand the functionalizing/hybridizing procedures, this paper begins with a brief description on synthetic methodologies of 2D nanomaterials. Subsequently, TMD nanosheets are used as typical models to demonstrate element doping, physical/chemical functionalization, and hybridization with other nanostructured materials for highlighting their new/improved properties, significant synergistic effects and novel/enhanced applications. Further, more heterostructures of diverse 2D nanomaterials are specifically summarized to emphasize their rational design and construction for promising applications. Finally, a summary is provided to acquaint the current research status and challenges ahead followed by perspectives in functionalized hybridization of 2D nanomaterials.

## 2 Strategic Fabrication of 2D Nanomaterials

**Figure 2:**
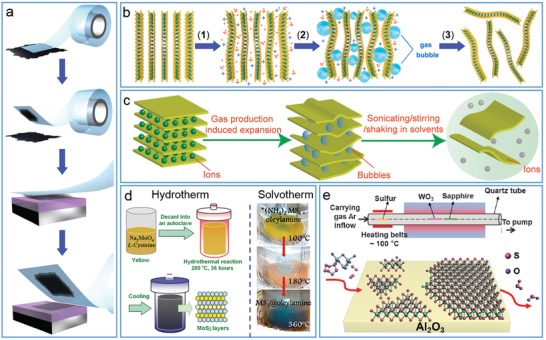
a–c) Schematic strategies for top‐down production of 2D nanomaterials. a) Mechanical cleavage by using Scotch tape. Reproduced with permission.^37^ Copyright 2012, Institute of Physics. b) Sonication‐driven exfoliation via 1) the addition of exfoliating/stabilizing agents, 2) sonication, and 3) increment of sonication time. Reproduced with permission.^38^ Copyright 2014, American Chemical Society. c) Ion intercalation‐assisted exfoliation. Reproduced with permission.^39^ Copyright 2018, The Royal Society of Chemistry. d) Schematic strategies for bottom‐up production of 2D nanomaterials: hydrothermal and solvothermal synthesis of MoS_2_ nanosheets. Reproduced with permission.^43^ Copyright 2015, The Royal Society of Chemistry; and with permission.^5^ Copyright 2011, American Chemical Society. e) Chemical vapor deposition of WS_2_ nanosheets. Reproduced with permission.^44^ Copyright 2013, American Chemical Society.

Most of 2D nanomaterials can be readily obtained through the exfoliation of their parent bulks, especially van der Waals layered materials via the so‐called top‐down approaches, which are mainly divided into mechanical cleavage (**Figure** **2**a), mechanical force‐driven (Figure 2b) and ion intercalation‐assisted exfoliation (Figure 2c) based on different driving forces.^22^, ^36^ Among them, the mechanical cleavage by using Scotch tape is the most straightforward route to fabricate high‐quality nanosheets onto substrates (e.g., SiO_2_/Si), but it is impossible for large‐scale production of nanosheets owing to the lack of sufficient scalability.^37^ In comparison, the liquid‐phase exfoliation driven by mechanical force or assisted by ion intercalation is more convenient for scalable production of nanosheets at high yield, providing an easier route to engineer the chemical and physical properties of nanosheets in solution phase. For example, sonication‐driven exfoliation is simply performed by directly sonicating parent materials in liquid after the addition of exfoliating agents (e.g., polymers, proteins) or stabilizing agents (e.g., surfactants) to facilitate the production of dispersed nanosheets,^38^ while ion intercalation‐assisted exfoliation is based on chemical or electrochemical ion intercalation in organic phase followed by exfoliation in aqueous solution through the generation of hydrogen gas.^39^ It was noted that ion intercalation‐based exfoliation is very effective to yield high concentration of single‐layer nanosheets, but the introduction/incorporation of small metal ions usually leads to structural and electronic deformations in TMD nanosheets different from their bulky counterparts (e.g., semiconductor MoS_2_ bulk in 2H phase is exfoliated into metallic nanosheets in 1T phase after Li‐intercalation).^40^

To increase productivity of 2D nanosheets at large quantity, layered intermediates with weaker interlayered interaction are often synthesized due to their easier and effective exfoliation into nanosheets. For example, graphite is first oxidized into graphene oxide (GO) in bulk as an intermediate followed by sonication‐driven exfoliation into GO nanosheets and further reduction to reduced GO (RGO) nanosheets for restoring sp^2^‐conjugated network and electrical conductivity,^41^ resulting in gram‐level production superior to other methods by directly using graphite. Also, layered hydrated WO_3_ (WO_3_·*n*H_2_O) or Bi_2_W_2_O_9_ intermediates are first synthesized and then exfoliated into WO_3_ nanosheets via mechanical cleavage or sonication‐driven method.^42^

In contrast, various nanosheets can be also synthesized via chemical reactions of special precursors under certain experimental conditions (i.e., the so‐called bottom‐up technique). Among them, wet‐chemical (e.g., hydrothermal and solvothermal) approaches are developed to produce a variety of nanosheets with controllable lateral size and layer thickness by choosing the proper precursors and optimizing the reaction conditions (Figure 2d), accompanied with the direct functionalization or hybridization of nanosheets via adding suitable functional components in reaction solution.^5^, ^43^ Alternatively, chemical vapor deposition (CVD) of precursors in gas phase is used to grow nanosheets on preselected substrates under high vacuum and high temperature (Figure 2e). It is very convenient to tune the size and thickness of 2D nanomaterials by controlling deposition time and gas flow rate, and further realize the hybridization of diverse 2D nanosheets at vertical and horizontal orientations.^44^, ^45^

## 3 Functionalized Hybridization of TMD Nanosheets

In the post‐graphene era, transition metal dichalcogenides in single and few layers have become more and more popular as emerging 2D nanomaterials because of their exceptional features and abundance in nature.^46^, ^47^, ^48^, ^49^, ^50^, ^51^, ^52^ In comparison with graphene that is a zero‐bandgap semiconductor and thus forms a bottleneck for its utilization in electronic devices and photoresponsive areas,^14^ TMD nanosheets have layer‐dependent bandgap from 0.8 to 2.4 eV in the infrared, near‐infrared, and visible region (e.g., monolayer and bulky MoS_2_ possess a direct bandgap of 1.9 eV and an indirect bandgap of 1.3 eV, respectively.^46^), robust spin–orbit coupling and superior electronic properties, and thus lead to their outstanding performance in energy‐harvesting, optoelectronics, and high‐end or flexible electronics.^47^, ^48^, ^49^ With a rapid progress in developing methodologies for readily preparing TMD nanosheets, a large number of important achievements have been achieved recently through element incorporation with ions/atoms, chemical/physical modification with small/macromolecules, and surface hybridization with functional nanostructures into/onto TMD nanosheets.^50^, ^51^, ^52^ In this section, we systematically present recent advances in the functionalized hybridization of TMD nanosheets and their improved properties/enhanced applications, which are reviewed in accordance with diverse approaches for introducing specific species into/onto TMD nanosheets (**Table** **1** shows the representative hybridized structures with specific applications and excellent performance).

**Table 1:** Summary on the hybridized 2D nanomaterials with other species and 2D nanomaterials

2D sheets	Functional species	Synthetic approaches to hybridized structures	Applications	Performances	Ref.
I: Hybridization of atoms, molecules, polymers, and nanostructures on TMD nanosheets					
TiS_2_	H atoms	Chemical exfoliation of Li‐intercalated TiS_2_ intermediate via a forced hydrogen incorporation process	HER	Enhanced electron concentration by 3 orders of magnitude than the bulk to reach an electrical conductivity of 6.76 × 10^4^ S m^−1^ at room temperature	58
MoS_2_	Hexadecanethiol	Covalent binding of alkanethiol to sulfur‐ vacancies on micromechanically exfoliated MoS_2_ nanosheets	FET	Drastic decrease in its source–drain current	67
MoS_2_	Polypyrrole	Chemical polymerization of pyrrole on MoS_2_ nanosheets	Supercapacitor	Specific capacitance of 700 F g^−1^ at the scan rate of 10 mV s^−1^, surpassing other systems of PPy materials	97
MoS_2_	CuInS_2_ QDs	Hydrophobic hybridization of dodecanethiol‐protected nanosheets and dodecanethiol‐capped QDs	HER	Great enhancement in photocurrent by 20% and 50% compared with pure QDs and pristine MoS_2_ nanosheets	110
WS_2_	CdS nanorods	Sonicating, evaporating and drying their mixed ethanol–water solution	HER	Substantial increment in HER rate by 26‐fold compared with CdS nanorods (no H_2_ evolution observed by using WS_2_ nanosheets alone)	111
MoS_2_	GaTe sheets	Vertical stacking of GaTe sheets on MoS_2_ nanosheets via van der Waals interaction to form p–n junctions	Photodetector	A rectification ratio of 4 × 10^5^, an external quantum efficiency of 61.68% and a photoresponsivity of 21.83 A W^−1^, a detectivity of 8.4 × 10^13^ Jones, much higher than commercial Si and InGaAs photodetectors	113
MoS_2_	TiO_2_ nanobelts	Hydrothermal growth of MoS_2_ nanosheets on TiO_2_ nanobelts by using sodium molybdate and thioacetamide	Photocatalytic degradation	Complete degradation of RhB (15 mg L^−1^) within 20 min, faster than pure TiO_2_ nanobelts or pure MoS_2_ nanosheets	127
II: Hybridization of metal and metal oxide nanoparticles on graphene					
Graphene	Au NPs	Successive electrodeposition of graphene and Au NPs on a glass‐carbon electrode	Biosensor	Improved conductivity/electrochemical sensing for attomolar Hg^2+^ detection after combining with single‐stranded DNA probes	145
Graphene	TiO_2_ NPs	Nucleation, growth, anchoring and crystallization of TiO_2_ on graphene during the chemical reduction of GO via a simple sol–gel strategy	Li^+^ battery	High specific capacity of ≈94 mA h g^−1^ at 59 C, twice as that of physically mixed composite	153
Graphene	ZnO NPs	Chemical reduction of GO mixed with ZnO by hydrazine followed by thermal evaporation	Photocatalytic degradation	Enhanced photocatalytic rate by ≈4 times compared to pristine ZnO in degradation of MB	156
Graphene	ZnO NPs	Hydrothermal treatment of ZnO and GO in water	Photocatalytic degradation	Fast degradation of deoxynivalenol (15 ppm) by 99% within 30 min, 3.1 times higher than that of pure ZnO	157
III: Hybridization of protons and semiconductor nanostructures on g‐C_3_N_4_ nanosheets					
g‐C_3_N_4_	Protons	Sonication‐driven exfoliation of g‐C_3_N_4_ in 10 m HCl	Biosensor	New heparin sensing platform with a detection limit of 18 ng mL^−1^	163
g‐C_3_N_4_	Co_2_P nanorods	Sonication‐driven embedding of Co_2_P nanorods into g‐C_3_N_4_ nanosheets	HER	High H_2_ production rate at 53.3 µmol g^−1^ h^−1^ (no H_2_ evolution observed by using g‐C_3_N_4_ alone)	170
g‐C_3_N_4_	BiPO_4_ nanorods	Electrostatic immobilization of BiPO_4_ nanorods on g‐C_3_N_4_ nanosheets	Photocatalytic degradation	Fast degradation of RhB by 94.3% within 6 min under UV light irradiation, ≈4.2 times and ≈1.5 times higher than BiPO_4_ and g‐C_3_N_4_, respectively	171
g‐C_3_N_4_	BiOCl NPs	Solvothermal synthesis of BiOCl in g‐C_3_N_4_ solution	Photocatalytic degradation	Fast removal of 4‐chlorophenol by 95% within 2 h, ≈12.5 and 5.3 times greater than pure BiOCl and g‐C_3_N_4_, respectively	177
g‐C_3_N_4_	TiO_2_ NPs	Hydrothermal calcination of mixed melamine and tetrabutyl titanate	Photocatalytic degradation	Fast degradation of RhB, 18.7 and 3.5 times better than that of pure TiO_2_ and g‐C_3_N_4_, respectively	179
g‐C_3_N_4_	TiO_2_ powder	Thermal calcination of mixed melamine and amorphous macro/mesoporous TiO_2_ powders	Photocatalytic degradation	Fast degradation of RhB up to 47.8 × 10^−3^ min^−1^, 7.2 and 3.1 times higher than pure TiO_2_ and g‐C_3_N_4_, respectively	180
g‐C_3_N_4_	ZnO, PANI	Chemical polymerization of aniline in the presence of g‐C_3_N_4_ and ZnO followed by thermal evaporation	Photocatalytic degradation	Fast degradation of MB and 4‐chlorophenol, 3.6 and 3.3 times higher than g‐C_3_N_4_, respectively	182
IV: Hybridization of BP and TMO nanosheets					
BP	Aryl diazonium	Covalent functionalization of aryl diazonium on BP nanosheets via phosphorus–carbon bonds	FET	Simultaneously improved the field‐effect transistor mobility and on/off current ratio	190
BP	AlO*_x_* overlayers	Hydrolytic deposition of AlO*_x_* layers by using trimethylaluminum in a Cambridge NanoTech reactor	FET	High on/off ratio (≈10^3^) and good mobility (≈100 cm^2^ V^−1^ s^−1^) for at least 2 weeks	194
BP	Ag NPs	Covalent linkage of Ag NPs on BP nanosheets by chemical reduction of AgNO_3_	Photocatalytic degradation	An enhancement up to ≈20‐fold in photodegradation of RhB compared to pristine BP nanosheets	198
BP	TiO_2_	Hydrolytic production of TiO_2_ in BP‐dispersed solution by using titanium isopropoxide	Photocatalytic degradation	High maintenance of photoactivity at ≈92% in photodegradation of RhB after 15 runs	199
BP	Zn_0.5_Cd_0.5_S	Sonication and centrifugation of mixed Zn_0.5_Cd_0.5_S and BP in absolute ethanol	HER	High H_2_ production rate of 137.17 mmol g^−1^ h^−1^, 5 times higher than Zn_0.5_Cd_0.5_S	202
BP	BiVO_4_	Electrostatic assembly of BiVO_4_ nanosheets on BP nanosheets	Photocatalytic water splitting	Increased photocurrent by 4.5 and 2.6 times compared with pure BP and BiVO_4_ to produce H_2_ and O_2_ at ≈160 and ≈102 mmol g^−1^ h^−1^	203
BP	ZnO nanowires	Mechanical exfoliation and transfer of BP sheets onto an already‐prepared ZnO nanowire	Photodetector	A high on/off ratio of ≈10^4^ in static rectification	209
CoO	Ni	Thermal exchange of ZnO nanosheets with cobalt chloride and nickel chloride in a furnace under nitrogen	Zinc–air battery	High discharge peak power density at 377 mW cm^−2^, small charge–discharge voltage of 0.63 V, stable working for >400 h at 5 mA cm^−2^	221
V: Interspecies hybridization of different 2D nanomaterials					
MoS_2_	Graphene	Gelation, reduction and self‐assembly of mixed MoS_2_ and GO nanosheets into a 3D porous structure	Li^+^ battery	Reversible capacity of 800 mA h g^−1^ at a current density of 100 mA g^−1^, and no capacity drop over 500 charge/discharge cycles at a current density of 400 mA g^−1^	240
MoS_2_	Graphene	Microscope‐assisted transfer of graphene onto MoS_2_ nanosheets	Photodetector	High responsivity at ≈1 × 10^10^ A W^−1^ at 130 K and ≈5 × 10^8^ A W^−1^ at room temperature	244
MoS_2_	Graphene	Hydrothermal treatment of sodium molybdate and GO assisted by L‐cysteine followed by annealing in H_2_/N_2_	Li^+^ battery	Highest specific capacity of ≈1100 mA h g^−1^ at a current of 100 mA g^−1^	246
MoSe_2_	Graphene	Alternative drop‐casting MoSe_2_ flakes and graphene on substrates	HER	High cathodic current density of 10 mA cm^−2^ at overpotential of 100 mV and high exchange current density of 0.203 µA cm^−2^	241
MoS_2_	g‐C_3_N_4_	Ultrasonication‐assisted coupling of MoS_2_ nanosheets into C_3_N_4_	Photocatalytic degradation	Photodegradation rate of RhB as high as 0.301 min^−1^, 3.6 times higher than that of bare C_3_N_4_	252
MoS_2_	BP	Mechanical exfoliation of BP sheets onto CVD‐grown MoS_2_ monolayer	Photodetector	Highest photodetection responsivity of 418 mA W^−1^, ≈100 times higher than other BP phototransistors and 26 times higher than WSe_2_ p–n diodes	254
MoS_2_	BP	Microscope‐assisted transfer of MoS_2_ sheet onto BP sheet to form a heterojunction in overlapped region	Photodetector	Fast microsecond response with the photoresponsivities of ≈22.3 and 0.1534 A W^−1^ at 532 nm and 1.55 µm, respectively	255
Graphene	g‐C_3_N_4_	Vacuum filtration approach to fabricate a flexible 3D hybrid film	HER	High exchange current density of 0.43 mA cm^−2^ and good durability with no loss of activity >5000 cycles	263
Graphene	g‐C_3_N_4_	Layer‐by‐layer assembly of graphene and g‐C_3_N_4_	Chemical sensor	Selective detection of NO_2_ as low as 100 ppb without light irradiation, and SO_2_ with a detection limit of 2 ppm under UV light irradiation	264
Graphene	g‐C_3_N_4_, CdS nanorods	Ultrasonication‐assisted formation of ternary CdS nanorods, g‐C_3_N_4_ and RGO nanosheets	HER	High H_2_ production rate of ≈4800 mmol g^−1^ h^−1^, 44, 11, and 2.5 times higher than those for C_3_N_4_, C_3_N_4_/RGO and C_3_N_4_/CdS, respectively	266
Graphene	MoO_3_	Thermal annealing of Mo‐MOFs mixed with GO nanosheets	Supercapacitor	Specific capacitance of 404 F g^−1^ at 0.5 A g^−1^ and a capacitance retention of ≈80% after 5000 cycles at 2 A g^−1^, comparable with other supercapacitors	267
Graphene	BP	Self‐assembly of BP–graphene sandwich structure in an argon‐filled glove box	Na^+^ battery	High specific capacity of 2440 mA h g^−1^ at a current density of 0.05 A g^−1^ with 83% capacity retained after operating for 100 cycles	269

### 3.1 Element Doping with Ions and Atoms

**Figure 3:**
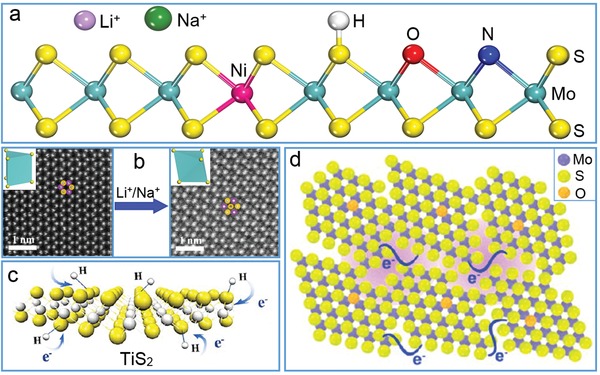
Element doping of TMD layers. a) Schematic doping of various ions and atoms into/onto MoS_2_ layers. b) Phase engineering of MoS_2_ nanosheet after intercalated by Li^+^ or Na^+^. Reproduced with permission.^54^ Copyright 2015, American Chemical Society; and with permission.^55^ Copyright 2014, American Chemical Society. c) TiS_2_ nanosheet doped with H. Reproduced with permission.^58^ Copyright 2013, American Chemical Society. d) MoS_2_ nanosheet doped with O. Reproduced with permission.^59^ Copyright 2013, American Chemical Society.

The intrinsic properties of TMDs are mainly determined by their atomic structures and imperfects in crystals, which renders the element doping into nanosheets very straightforward to tune their electronic and optical properties via the change in band alignment, and/or endow magnetic functions via the introduction of special elements.^53^ As one of the most investigated 2D nanomaterials, few layered MoS_2_ nanosheets have been extensively doped with ions and atoms to regulate their structures and properties (**Figure** **3**a). For example, small metal ions such as Li^+^ and Na^+^ can intercalate into the interlayers of MoS_2_ nanosheets or bind on their surface via electrostatic interaction. As known, naturally occurred MoS_2_ is found to be hexagonal (2H) phase with semiconducting properties, whereas chemical intercalation by Li^+^ induces a phase change to trigonal (1T) phase with metallic properties.^54^ In Wang's work, the phase transition and occupancy of the intercalated Na^+^ were directly clarified by using aberration‐corrected scanning transmission electron microscope (Figure 3b). A critical point for the structural evolution at the Na/Mo ratio of >1.5 was revealed to enrich the understanding of phase transition and intercalation chemistry for MoS_2_.^55^ Lei et al. reported a general strategy for ion‐functionalization on TMD nanosheets without structural alteration by a Lewis acid–base reaction on lone pair electrons.^56^ Typically, Ti^4+^ ions were readily coordinated onto a n‐type InSe layer through the formation of a p‐type [Ti^4+^ *_n_*(InSe)] complexes, and this approach is also applicable for coordinating other metal ions such as B^3+^, Al^3+^, and Sn^4+^ and on other 2D nanosheets such as MoS_2_ and WS_2_. Liu's group reported another general way to dope metal ions such as Gd^3+^, Fe^3+^, Co^2+^, Ni^2+^, and Mn^2+^ into WS_2_ nanosheets by the chemical reaction of WCl_6_, MCl*_x_*, and sulfur at 300 °C in the presence of oleylamine and 1‐octadecene.^57^ It was also reported that Nb‐doped MoS_2_ became p‐type from n‐type MoS_2_ before doping while Mn‐doped MoS_2_ exhibits long‐range ferromagnetic behavior.^21^

Besides the metal ions, nonmetal atoms such as H, O, and N were also incorporated into TMD nanosheets to achieve special properties and enhanced applications. Xie's group reported hydrogen‐modified TiS_2_ nanosheets (Figure 3c) and oxygen‐incorporated MoS_2_ nanosheets (Figure 3d) with high electrical conductivity toward efficient hydrogen evolution reaction (HER), which were obtained by exfoliating Li‐intercalated TiS_2_ intermediate via a forced hydrogen incorporation process^58^ and controlling MoS_2_ crystallization process at lower temperature,^59^ respectively. The introduced hydrogen on TiS_2_ layers donated extra electrons to layered TiS_2_ framework for enhancing its electron–electron correlations and conductivity by three orders of magnitude. The existence of oxygen in MoS_2_ offered abundant unsaturated sulfur atoms while improved its conductivity via controllable disorder engineering. Recently, the doping of nitrogen in MoS_2_ nanosheets was also realized by a modified one‐step sintering approach.^60^ Revealed by density functional theory (DFT) calculations, the active HER sites were identified at the edges of nitrogen‐doped MoS_2_ and the conducting charges spread over nitrogen‐doped basal plane owing to strong orbital hybridizations of Mo 3d, S 2p, and N 2p at Fermi level, which enhanced the conductivity of MoS_2_ sheets for promoting charge transfer in the efficient HER. Further, a surface modulation approach was developed by decorating isolated nickel atoms onto the basal plane of MoS_2_ sheets by annealing at 600 °C in a flow of Ar/H_2_ for boosting hydrogen evolution activity, and this brings about more opportunities for enhancing the intrinsic catalytic activity for electrocatalytic water splitting and other energy‐related reactions.^61^

### 3.2 Surface Modification with Organic Molecules

**Figure 4:**
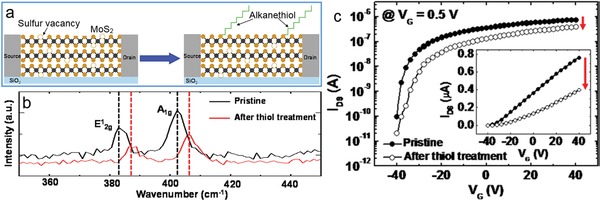
Sulfur vacancy passivation by alkanethiol molecules. a) Schematic drawings of a MoS_2_ field effect transistor before and after the treatment with alkanethiol molecules. b) Raman spectra of sulfur vacancies. c) *I*_DS_–*V*_G_ curves before and after thiol treatment with logarithmic scale. The inset shows *I*_DS_–*V*_G_ curves with linear scale. Reproduced with permission.^67^ Copyright 2015, American Chemical Society.

Surface modification on TMD nanosheets with organic molecules through thiol chemistry reaction, C—S bonding linkage and noncovalent interactions not only improves their electrical and optical properties, but also manipulates their solubility, compatibility to other moieties, and hybridizing ability with other functional materials.^21^ As reported, thiol ligand modifications can readily occur on both internal and perimeter edges of MoS_2_ nanosheets due to their higher molecular affinities.^62^, ^63^ For example, Huang and co‐workers modulated the zeta‐potential and colloidal stability of MoS_2_ nanosheets via their edge conjugation with various thiol‐terminated ligands HS(CH_2_)_11_OCH_2_(CH_2_OCH_2_)_3_CH_2_—R (R=OH, COO^−^, and NMe_3_^+^), and the surface modified MoS_2_ nanosheets were further used as protein receptors for binding β‐galactosidase.^64^ Similarly, other thiol‐terminated ligands such as mercaptopropionic acid, 1‐thioglycerol and L‐cysteine were also chemically conjugated on the edges of MoS_2_ and WS_2_ nanosheets during their exfoliation process.^65^ In addition, disulfide ligands such as lipoic acid were covalently linked on the edge defects of MoS_2_ nanosheets via thiol chemistry reaction.^66^ Recently, Lee's group reported the covalent binding of sulfur‐containing groups on the surface sulfur vacancies of micromechanically exfoliated MoS_2_ (**Figure** **4**a), as indicated by the Raman spectral change of the surface‐modified MoS_2_ films (Figure 4b).^67^ After the treatment with alkanethiol, the MoS_2_ field effect transistors (FETs) exhibited an impressive change in electrical and optical characteristics (Figure 4c). Following the similar process, Yu et al. repaired the sulfur vacancies of MoS_2_ nanosheets by using 3‐mercaptopropyltrimethoxysilane to improve their interface for great reduction in charged impurities and traps.^68^ In general, the amount of the attached thiol molecules can be simply adjusted by optimizing experimental conditions, rendering excellent structural stability and high chemical reactivity for further use.

**Figure 5:**
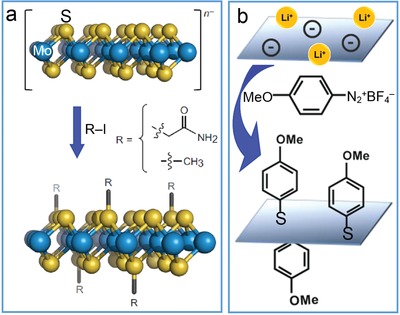
C—S bonding‐mediated surface functionalization of MoS_2_ nanosheets. a) Schematic modification of MoS_2_ nanosheets with 2‐iodoacetamide or iodomethane. Reproduced with permission.^69^ Copyright 2014, Nature Publishing Group. b) Schematic basal‐plane functionalization of MoS_2_ with 4‐methoxyphenyldiazonium tetrafluoroborate. Reproduced with permission.^70^ Copyright 2015, American Chemical Society.

As demonstrated above, the attachments (i.e., binding reactions) of functional groups on TMD nanosheets mainly occur at defect sites including edge sites and sulfur vacancies, and often become unfavorable due to the limited amount of defect sites. To this end, Chhowalla's group reported covalent modification of TMD sheets with 2‐iodoacetamide or iodomethane directly on chalcogen atoms via the formation of C—S bonds due to electron transfer between the electron‐rich metallic 1T phase and the added organohalide agent (**Figure** **5**a).^69^ The covalent attachment enabled the 1T phase to exhibit semiconducting property instead together with tunable strong photoluminescence and gate modulation in FETs. Similar approach was also developed by Backes's group through quenching the negative charges residing on MoS_2_ nanosheets by electrophiles such as diazonium salts (Figure 5b).^70^ Meanwhile, basal‐plane functionalization of MoS_2_ nanosheets was realized via the coordination of surface sulfur atoms of MoS_2_ with M(OAc)_2_ salts (M = Ni, Cu or Zn; OAc = acetate) for higher stability and better dispersion in solvents.^71^

Besides the covalent functionalizations, noncovalent interactions have been frequently adapted for hybridizing TMD nanosheets with organic molecules. For instance, ammonium tetrathiomolybdate and ammonium tetrathiotungstate were used to synthesized MoS_2_ and WS_2_ nanosheets in the presence of oleylamine and the resulting surface binding of oleylamine played a dynamic protective function to obtain free‐standing nanosheets with high stability.^5^ Interestingly, the layer number of resulting nanosheets was controlled by using different capping ligands. In Jung's work,^6^ single‐layer WSe_2_ nanosheets were grown by using tungsten hexacarbonyl and diphenyl diselenide as precursors in the presence of oleic acid while multilayer nanosheets were produced in the presence of oleylalcohol or oleylamine. As understood by simulation with DFT, there is a stronger binding affinity of oleylalcohol or oleylamine on WSe_2_ edges, and thus WSe_2_ nanosheets may grow perpendicularly to form multilayer nanosheets. As there is a weaker binding affinity of oleic acid on WSe_2_ edges, and thus WSe_2_ nanosheets may extend their growth in plane to form larger single layer nanosheets. In McDonald's work, it was found that MoS_2_ facilitated the oxidation of thiol ligands into disulfide ligands accompanied with the corresponding change from surface coordination of thiol to surface physisorption of disulfide on MoS_2_ nanosheets.^72^ It is known that three layers of atoms are displayed in a MoS_2_ monolayer by sandwiching a transition metal layer between two chalcogen layers.^18^ As such, organic thiols can easily coordinate on the freshly cleaved sulfur‐vacancies of MoS_2_ nanosheets, as reported for the mechanically exfoliated MoS_2_ nanosheets.^67^, ^68^ For the liquid phase‐exfoliated MoS_2_ nanosheets, in contrast, the surface physisorption of resulting disulfide predominantly occurs on nanosheets instead through hydrophobic interaction as described in our publication.^73^

Alternatively, sodium cholate, hemin, and pyrene were also noncovalently functionalized on TMD nanosheets via van der Waals force for improving dispersion stability, intrinsic peroxidase‐like activities and electrical properties.^74^, ^75^, ^76^ Meanwhile, a thermo‐responsive polymeric ionic liquid was similarly used for high‐efficiency exfoliation and noncovalent modification of MoS_2_ nanosheets, resulting in thermo‐responsive phase transition to produce a colored hydrogel with dual temperature‐ and photoresponsive functions.^77^ Furthermore, the noncovalent functionalization was extended by using optoelectronically active chromophores (i.e., free‐base phthalocyanine) to fabricate electronic heterostructures with enhanced optoelectronic and exploitable charge transfer properties.^78^ Additionally, a perylene‐diimide derivative was hybridized on MoS_2_ nanosheets to prepare large‐area heterojunction films, exhibiting a sixfold improvement in photocurrent through the transfer of photogenerated holes between MoS_2_ and perylene‐diimide.^79^

### 3.3 Surface Adsorption with Macromolecules

Surface hybridization of macromolecules on TMD nanosheets can effectively improve the stabilization/dispersion of nanosheets in solution^80^ and biocompatibility in biological applications such as biomedicine and photothermal therapy (PTT).^81^, ^82^ When the anchored macromolecules carry additional functional groups, the modified TMD nanosheets become chemically reactive to facilitate their usage in sensitive and selective detection.^83^ Moreover, the existence of macromolecules in exfoliating solution may greatly increase the yield of nanosheets through optimizing their surface energy, and the layer thickness is controlled through changing the type or concentration of macromolecules.^73^ Until now, a variety of macromolecules were successfully bonded on TMD nanosheets via covalent or noncovalent interactions.^84^ Compared with covalently binding sulfur‐containing macromolecules including synthetic and biopolymers via organosulfur reaction,^85^ noncovalent hybridization of TMD nanosheets with macromolecules were realized more simply and conveniently during the preparation or exfoliation of nanosheets.

Synthetic polymers such as polyvinylpyrrolidone (PVP) and polyethylene glycol (PEG) were directly employed to produce the hybridized TMD nanosheets in polymer solution via polymer‐assisted exfoliation or chemical synthesis. Through simultaneous exfoliation and noncovalent bonding with PVP, the resultant PVP‐modified MoSe_2_ nanosheets were demonstrated to be a promising candidate for PTT agent and were also encapsulated into a hydrogel matrix as intelligent devices.^86^ With various wet‐chemical methods, PEG molecules were successfully coated on various TMD nanosheets including MoS_2_, TiS_2_, and ReS_2_ nanosheets.^87^, ^88^, ^89^, ^90^ For example, PEGlated MoS_2_ nanosheets were synthesized hydrothermally in the presence of (NH_4_)_2_MoS_4_ to achieve a good photothermal conversion performance and excellent colloidal/photothermal stability for photothermal therapeutics of cancers without exhibiting hemolysis, coagulation, and toxicity.^87^ To further improve the therapeutic efficacy, MoS_2_‐PEG nanosheets were integrated with poly(lactic‐*co*‐glycolic acid) and doxorubicin in a drug delivery implant for near‐infrared‐triggered synergistic tumor hyperthermia without diffusing into the circulation of body fluids.^88^ In Liu's work, lipoic acid was first grafted on PEG and then coated on exfoliated MoS_2_ nanosheets via thiol reaction for producing nanocarriers to effectively load therapeutic molecules while maintaining excellent physiological stabilities.^91^

Mixed synthetic polymers or block copolymers were also used to functionalize TMD nanosheets for better applications in catalysis and energy storage. For example, poly(3,4‐ethylene dioxythiophene):poly(styrene sulfonate) (PEDOT:PSS), an electrostatically attracted mixture of two ionomers was employed to exfoliate WS_2_ nanosheets as a hole extraction layer for boosting performance of organic solar cells caused by their island‐like morphology and benzoid–quinoid transition.^92^ Triblock copolymer poly(ethylene glycol)‐poly(propylene glycol)‐poly(ethylene glycol) (PEG‐PPG‐PEG, known by its trade name as P123) was used to produce MoS_2_ nanosheets with a strong photoluminescence in visible region.^93^, ^94^ Triblock polymer poly(ethylene oxide)‐poly(propylene oxide)‐poly(ethylene oxide) (PEO‐PPO‐PEO, known by its trade name as Pluronic) was utilized to produce highly concentrated solution of MoS_2_ nanosheets while its unimers rather micelles were the optimal form to achieve the highest yield of exfoliation and the thinnest thickness of layers.^95^ Recently, diblock copolymer poly(*N*‐isopropylacrylamideco‐ionic liquid) with additional functions (e.g., thermo‐responsive properties) was modified on MoSe_2_ nanosheets to form a multifunctional nanocomposite.^96^ Alternatively, the adsorption and subsequent polymerization of pyrrole were carried out on MoS_2_ nanosheets to construct high performance electrodes of supercapacitors.^97^

**Figure 6:**
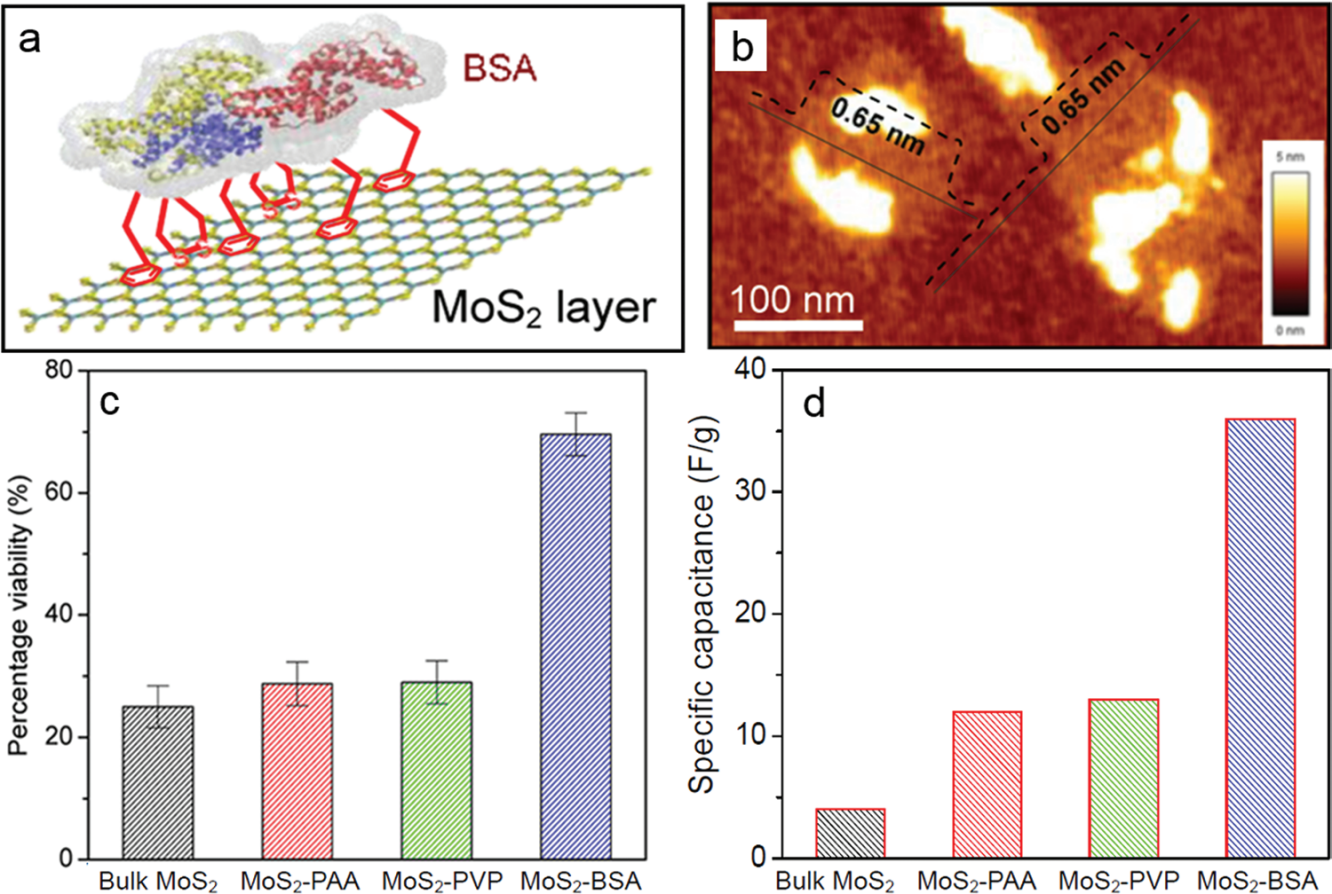
Noncovalent hybridization of BSA on MoS_2_ nanosheets. a) Schematic binding of BSA on MoS_2_ layer via benzene rings and disulfides. b) AFM image of single‐layer MoS_2_ bonded with BSA. c) Biocompatibility and d) specific capacitance of MoS_2_‐BSA nanosheets and other MoS_2_ products. As observed, the surface‐hybridized BSA endows a better biocompatibility and a larger specific capacitance due to its biological and insulative nature. Reproduced with permission.^73^ Copyright 2015, American Chemical Society.

**Figure 7:**
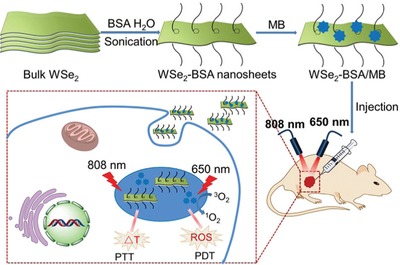
Schematic illustration of BSA‐induced exfoliation of WSe_2_ nanosheets and then loading with photosensitizer methylene blue on nanosheets for synergistic photothermal/photodynamic cancer therapy. Reproduced with permission.^99^ Copyright 2017, The Royal Society of Chemistry.

Meanwhile, biopolymers such as proteins with rich functional groups and good biocompatibility are ideal ligands to functionalize TMD nanosheets for sensing, biological, and medicine applications. In our previous work, as the first biomacromolecule, bovine serum albumin (BSA) via its nonpolar groups such as benzene rings and disulfide groups was firmly bonded on TMDs (e.g., MoS_2_, WS_2_, and WSe_2_) through their strong hydrophobic interaction (**Figure** **6**a,b).^73^ As such, BSA served as an effective exfoliating agent under ultrasonication to result in BSA‐stabilized TMD nanosheets with greatly improved biocompatibility (Figure 6c) and much larger capacitance for sensing applications (Figure 6d) due to the successful hybridization of BSA on MoS_2_ nanosheets. These outstanding features render BSA‐stabilized TMD nanosheets a star materials in drug delivery system and tumor therapy.^98^ As reported, the WSe_2_‐BSA nanosheets were employed as efficient photothermal moieties and photosensitizing agent carrier for photodynamic therapy (**Figure** **7**).^99^ The dual mode of photothermal and photodynamic therapy possessed a great synergistic effect to significantly improve therapeutic efficacy for cancer cells.^100^ Additionally, MoS_2_‐BSA sheets were also used as promising lubricating additive to improve friction‐reducing and anti‐wearing performance at a relatively low concentration.^101^ Besides BSA, other proteins such as thionin,^102^ hemoglobin,^101^ lysozyme,^103^ and silk fibroin^104^ were also utilized to simultaneously exfoliate and hybridize TMD nanosheets. Most of products possessed few layered nanostructures due to weaker noncovalent interaction. Interestingly, the as‐processed silk fibroin has a low efficiency in exfoliating MoSe_2_ nanosheets, but carboxyl‐modified silk fibroin can remarkably improve the exfoliation efficiency to achieve a high‐yield production of nanosheets.^104^ Moreover, the MoSe_2_‐fibroin nanosheets showed wound disinfection and healing efficacy in a rapid and effective way under a low‐dose of H_2_O_2_, benefiting from the synergistic effect of excellent peroxidase‐like activity of MoSe_2_ sheets and biorepairing ability of silk fibroin.

Similarly, other biopolymers such as chitosan, sodium alginate, and nucleotides were also reported to bind on MoS_2_ nanosheets via noncovalent interaction to facilitate exfoliation and prevent re‐assembling process.^105^, ^106^, ^107^, ^108^, ^109^ Chitosan exfoliated and decorated MoS_2_ nanosheets effectively as a biocompatible near‐infrared material in photothermal ablation of cancer.^106^ Sodium alginate was found to be very efficient to produce an unprecedented concentration of WS_2_ nanosheets originated from strong interaction of hydrogen bonding and coordination between sodium alginate and WS_2_ layers.^109^ In Choi's work, the high‐yield production of WS_2_ and WSe_2_ nanosheets was facilely achieved by using single‐stranded DNA with high molecular weight,^105^ which enabled a high stabilization of resulting nanosheets via the electrostatic repulsion between sugars of DNA backbone. Recently, Ayán‐Varela et al. reported the adsorption of DNA/RNA nucleotides on TMDs to exfoliate nanosheets through Lewis acid–base type interactions between nucleotides and nanosheets.^106^

### 3.4 Surface Hybridization with Other Nanostructures

Through surface hybridization of TMD nanosheets with other functional nanostructures, novel or enhanced properties are synergistically achieved arising from two or multiple components in the resultant composites.^34^ As reported, after combining TMD nanosheets with metal, oxide, sulfide and carbon‐based nanostructures, the resulting photocatalytic and electrocatalytic activities were significantly improved to enhance their applications in plasmonics, photocatalysis, photovoltaics, and photodetections.^34^, ^35^ Typically, metal nanoparticles are successfully hybridized at the dangling bond‐rich edges of MoS_2_ nanosheets via nonliquid exfoliation or at the defective basal planes of MoS_2_ nanosheets via direct reduction of metal salts in the presence of surfactants or stabilizers, and these were summarized in previous review papers.^28^, ^34^ Here we outline the recent achievements on the surface hybridization of TMD nanosheets with different nanostructures together with the surface hybridization of various nanostructures with TMD nanosheets for improving properties and applications.

A variety of nanostructures were physically adsorbed onto TMD nanosheets by using wet‐chemistry approaches. In Srivastava's work, dodecanethiol‐protected MoS_2_ nanosheets and dodecanethiol‐capped CuInS_2_ quantum dots were simply mixed together to produce hybrids via their hydrophobic interaction, exhibiting an efficient charge separation and transfer between quantum dots and nanosheets with 20% and 50% enhancement in photocurrent compared with pure quantum dots and pristine MoS_2_ nanosheets_._ 110 After mixing CdS nanorods with WS_2_ nanosheets in ethanol‐water solution, WS_2_/CdS hybrids were obtained through subsequent sonication, evaporation and drying under vacuum to achieve up to 26‐fold increment in hydrogen evolution rate compared to CdS nanorods. It is noted that hydrogen evolution was not observed by using WS_2_ nanosheets alone.^111^ Also, the mixed solution of Au nanoparticles and MoS_2_ nanosheets was incubated to assemble them into hybrids, and further drop‐casted on gold electrode after removing the nonassembled ones by centrifugation to show higher current density/electron mobility and faster mass transport.^112^ In addition, GaTe sheets were vertically stacked on MoS_2_ nanosheets via van der Waals interaction to form p–n junctions.^113^ The heterostructure of p‐GaTe/n‐MoS_2_ exhibited an excellent photovoltaic and photodetecting performance, including a rectification ratio of 4 × 10^5^, an external quantum efficiency of 61.68% and a photoresponsivity of 21.83 A W^−1^. Also, a detectivity of 8.4 × 10^13^ Jones was achieved, much higher than commercial Si and InGaAs photodetectors. Recently, different sized graphene quantum dots (GQDs) were successfully deposited on CVD‐grown MoS_2_ nanosheets via pipetting or spin‐coating to form van der Waals heterostructures, arousing electron transfer from GQD to MoS_2_ layer and cascade relaxation dynamics.^114^

**Figure 8:**
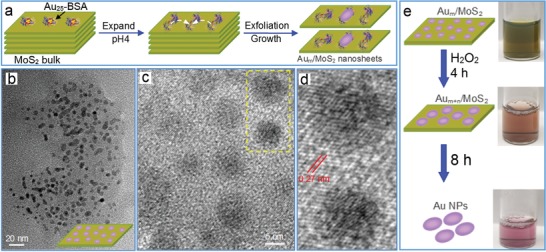
Epitaxial growth of Au nanoparticles on MoS_2_ nanosheets by using BSA‐caged Au_25_ nanoclusters. a) Schematic exfoliation of MoS_2_ nanosheets and subsequent surface growth of Au_25_ clusters into Au*_m_* nanoparticles. b) Low‐resolution and c) high‐resolution TEM images of Au*_m_*/MoS_2_ nanosheets, and d) their enlarged TEM image from the highlighted area in (c). e) Schematic growth of Au*_m_* to Au*_m_*_+_ *_n_* nanoparticles on MoS_2_ nanosheets with time upon the addition of H_2_O_2_, and the corresponding color evolution. Reproduced with permission.^122^ Copyright 2018, The Royal Society of Chemistry.

Through epitaxial growth, air‐annealing, thermal calcination and hydrothermal treatment, various nanostructures were chemically bound onto TMD nanosheets in gas phase or liquid phase. For example, gold nanocrystals were conveniently deposited on single‐layer MoS_2_ film by using a thermal evaporator for catalytic activation of CO and other small molecules.^115^ More interestingly, Fang's group used electron beam lithography and thermal evaporation method to fabricate various gold structures on CVD‐grown MoS_2_ monolayers, such as nanodisks,^116^ spiral ring,^117^ chiral pattern,^118^ grating array,^119^ and nanoantenna.^120^ Similarly, the designed silver nanodisks were also prepared on WS_2_ monolayers to achieve a large Rabi splitting (≈300 meV) at ambient conditions.^121^ Alternatively, our group developed a solution‐phase approach for growing size‐controlled Au nanoparticles on ultrathin MoS_2_ sheets by employing BSA‐caged Au_25_ nanoclusters as both exfoliating agent and gold precursor.^122^ As indicated in **Figure** **8**a, the conformational expansion of BSA on MoS_2_ surface via their hydrophobic interaction induced the effective exfoliation of MoS_2_ nanosheets under sonication, and this expansion also expose Au nanoclusters so as to facilitate their epitaxial growth into Au*_m_* nanoparticles (≈5 nm) on MoS_2_ nanosheets (Figure 8b–d). Upon the addition of H_2_O_2_, the Au*_m_* nanoparticles were further grown on nanosheets to tune the size in a range of 5–30 nm (Figure 8e). With their synergistic effect, Au*_m_*/MoS_2_ hybrids possessed an outstanding photocatalytic capability in degradation of methylene blue, much higher than that of individual components and their mixed product. Recently, ultrafine TiO_2_ nanoparticles were anchored on few layered MoS_2_ nanosheets by hydrolyzing tetrabutyl titanate on MoS_2_ nanosheets and further calcining at high temperature, which promoted the accessibility of electrolyte to 2D interlayer space and thus exhibited a remarkably improved lithium storage capacity.^123^ Also, WO_3_ nanoparticles were functionalized on WS_2_ nanosheets through air‐annealing at appropriate conditions for facilitating charge separation of photogenerated electron–hole pairs via charge transfer.^124^ Further, nickel hydr(oxy)oxide nanospheres were hybridized onto 1T‐phase MoS_2_ sheets through the chemical reaction of NiCl_2_ with NH_4_HCO_3_ in an ethanol solution for remarkably enhancing the HER activities in neutral and alkaline conditions.^125^ In other examples, ZnS particles were synthesized on MoS_2_ nanosheets through a two‐step hydrothermal strategy to broaden their sensing range from near‐infrared to ultraviolet region,^47^ while Ag_3_PO_4_ nanoparticles were coated on MoS_2_ nanosheets via an organic phase strategy for achieving higher photocatalytic activity.^126^

**Figure 9:**
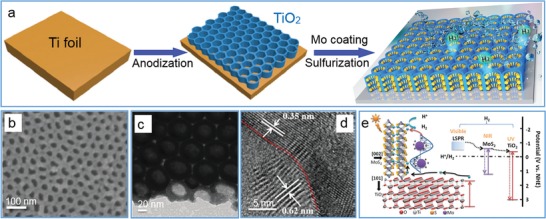
The hierarchical growth of MoS_2_ nanosheets on the inner surface of TiO_2_ nanocavities. a) Schematic illustration for the fabrication process of MoS_2_@TiO_2_ heterostructures. b) Top‐view SEM image, c) TEM image and d) high‐resolution TEM image of MoS_2_@TiO_2_ heterostructure with a MoS_2_ thickness of 10 nm. e) Schematic diagram of the energy band structure, plasmonic resonance, and electron transfer pathway in the MoS_2_@TiO_2_ heterojunction. Reproduced with permission.^129^ Copyright 2018, The Royal Society of Chemistry.

On the contrary, TMD nanosheets were grown on various nanostructures as well. For example, MoS_2_ nanosheets were coated on TiO_2_ nanobelts with inhibited growth along *c*‐axis by hydrothermal treatment of sodium molybdate and thioacetamide in the presence of TiO_2_ nanobelts, and the resultant TiO_2_@MoS_2_ heterostructures showed a higher photocatalytic ability in H_2_ production and better performance in photocatalytic degradation of dye molecules.^127^ Similarly, MoS_2_ nanosheets were fabricated on TiO_2_ nanobelts after hydrothermal treatment of ammonium tetrathiomolybdate with TiO_2_ nanobelts to harvest both ultraviolet and visible light for enhancing photocatalytic property due to the promoted separation of photoinduced carriers.^128^ Through sequential anodization of titanium foil in HF/H_3_PO_4_, e‐beam evaporation of Mo and CVD sulfurization, highly ordered honeycomb‐shaped arrays of TiO_2_ nanocavities were first obtained and successively deposited with Mo on the inner surface of arrays followed by sulfurization to form MoS_2_ nanoflakes in 3D hierarchical configuration (**Figure** **9**a–d).^129^ The resulting MoS_2_@TiO_2_ architecture in hierarchical configuration exhibited a full‐solar‐spectrum absorption and excellent photocatalytic ability for hydrogen evolution, profiting from the improved charge‐carrier separation by synergistic plasmonic effect and enhanced conductivity of “hot” electrons in the highly ordered architecture (Figure 9e). Further, MoS_2_ nanosheets were chemically synthesized on N‐doped carbon nanowall arrays to fabricate MoS_2_‐based hierarchical architecture as an efficient sodium ion battery anode.^130^

## 4 Representative Hybridization of Other 2D Nanomaterials

Accompanied with the extensive exploration on functionalized hybridization of TMD nanosheets, new and important achievements have also been gained in recent decades to hybridize special species on other 2D nanomaterials, including graphene, graphitic carbon nitride, black phosphorus, and transition metal oxide nanosheets. According to their different surface properties and expected functions after hybridization, a variety of effective functionalization methods have been rationally established to address specific objective for utilizing these 2D materials in promising applications. This section representatively describes various hybridization strategies and improved properties of more 2D hybrids beyond TMD's ones as summarized in last section.

### 4.1 Hybridization of Graphene

The synergistic hybridization of graphene with different materials can optimize its functions and properties for improving its performance in various fields such as electrochemistry, analytical chemistry and catalysis.^131^ Meanwhile, the surface modification or adsorption of organic ligands on graphene can be realized conveniently and effectively by using GO nanosheets as an intermediate due to their rich functional groups (e.g., hydroxyl, carboxyl, and epoxide).^41^ In this area, Dong's group overviewed the molecular engineering and hybridization of graphene with metal, metal oxides, quantum dots and carbon nanotubes,^132^ while Kim's group summarized various graphene‐based composites including polymers, carbon nanomaterials and inorganic nanoparticles.^133^ Here, we outline the recent research progress and important achievements to update this dynamic research field (Table 1).

#### 4.1.1 Carbon Nanostructures

With the same chemical element, carbon materials are often hybridized with graphene to improve its performance in various applications. For example, Shi et al. grew graphene in the voids of ultrathin carbon nanotube film on copper by CVD method for enhancing their strength and load transfer capabilities.^134^ Further, nanostructured porous carbon was hybridized with graphene and carbon nanotubes to form hierarchical all‐carbon architectures with interconnected micro/mesopores, which exhibited an extraordinary electrical conductivity for being utilized in lithium–sulfur batteries.^135^ When the current rate at 0.5 C, the composite cathode had an ultrahigh specific capacity of 1121 mA h g^−1^ and an impressive cycling stability within 150 cycles. Recently, 1‐pyrenebutyric acid‐modified γ‐cyclodextrin was assembled on folic acid‐functionalized graphene via π–π interaction for hosting C_60_ into γ‐cyclodextrin via host–guest chemistry, and the resulting graphene/C_60_ hybrids exhibited an excellent tumor‐killing efficiency in photodynamic and photothermal therapy, superior to other reported graphene/C_60_ systems.^136^

#### 4.1.2 Nickel Nanostructures

Ni nanoparticles were decorated on RGO nanosheets via high temperature reduction of NiCl_2_ and GO by H_2_ to exhibit much higher electrocatalytic and dye‐sensitized activities in HER than individual Ni and RGO counterparts.^137^ The defect‐anchored nucleation and spatially confined growth were also used to synthesize Ni–Fe layered double hydroxide on graphene with an excellent performance in oxygen evolution reactivity, outperforming commercial Ir/C catalysts.^138^ Meanwhile, the heterostructure of Ni–Al layered double hydroxide and graphene was fabricated by self‐assembly to construct high‐performance asymmetric supercapacitor with a better cycling stability and a higher power density, resulting from more stable structure, more active sites and faster ion diffusion in the heterostructures.^139^ In addition, ternary Ni_2−_ *_x_*Co*_x_*P nanocrystals were similarly hybridized with RGO to activate the surface sites and promote the charge transfer for boosting catalytic activity.^140^

**Figure 10:**
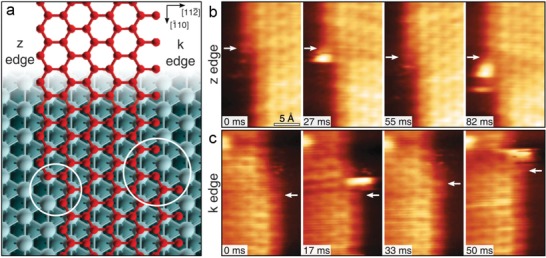
Graphene growth along Zigzag (z) and Klein (k) edges on Ni(111). a) Zigzag and Klein edges of a top‐fcc epitaxial graphene layer. b,c) High‐speed scanning tunneling microscopy acquired in quasi‐constant height mode at the z edge b) and the k edge c). White arrows indicate the position of C atoms in fcc‐hollow sites near the kink. Reproduced with permission.^143^ Copyright 2018, Science.

Based on the orbital theory, the chemical interaction between Ni and graphene is relatively strong through hybridizing the d‐electrons of Ni and the p‐orbitals of graphene, and this is certified by a much smaller distance between the Ni surface and graphene compared to the one between graphitic layers (0.21 vs 0.33 nm).^141^ With a matched lattice (246 vs 249 pm) as well, graphene can readily grow on Ni(111) as a desired substrate via physical vapor deposition.^142^ A recent research studied the catalytic role of single metal adatoms during the growth of graphene on Ni substrate.^143^ As shown in **Figure** **10**, the catalytic action of individual Ni atoms at the edges of a growing graphene flake was directly captured by scanning tunneling microscopy with a time resolution down to milliseconds, and further rationalized by force field molecular dynamics and DFT calculations.

#### 4.1.3 Gold and Silver Nanostructures

Au nanoclusters were introduced onto GO nanosheets via electrostatic interaction to exhibit excellent peroxidase‐like activity in a wide range of pH.^144^ More typically, graphene and Au were successively electrodeposited on a glass‐carbon electrode (GCE) surface to build electrochemical sensors with improved conductivity for attomolar Hg^2+^ detection after combined with single‐stranded DNA probes.^145^, ^146^ Similarly, Au nanorods was also hybridized with graphene to form a film on GCE as an electrode for developing electrochemical and electro‐chemiluminescence (bio)sensors.^147^, ^148^ Alternatively, graphene was employed to induce the nucleation and growth of Au for synthesizing single‐crystalline Au nanobelts with a preferable (111) orientation on graphene.^149^ The shapes and structures of the nanobelts were highly tunable through changing the interfacial interaction between Au atoms and the graphene lattice via surface modification, and the resulting hybrids exhibited extraordinary detection sensitivity when serving as a flexible surface‐enhanced Raman scattering (SERS) substrate.

Similar to the growth of Au nanoclusters to nanoparticles,^122^ Ag nanoclusters were successfully deposited on graphene and then agglomerated into Ag nanodots of 2–3 nm.^150^ In addition, Ag/AgX (X = Br, Cl) nanoparticles was enwrapped into GO architecture to form hybrids via a water/oil system and displayed distinctly enhanced photocatalytic activities through facilitating charge transfer and suppressing the recombination of electron–hole pairs.^151^ In another work, RGO was first coated on the surface of AgBr nanoparticles followed by incorporation into graphene to form hydrogels with 3D network structure.^152^ The dual hybridization promoted the rapid migration and separation of photogenerated charges for significantly improving pollutant removal efficiency.

#### 4.1.4 Metal Oxides

**Figure 11:**
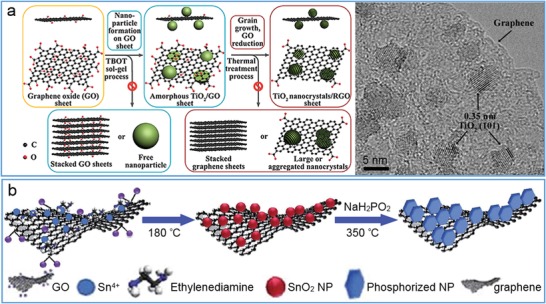
Hybridization of metal oxides on RGO nanosheets. a) Schematic representation of the sol–gel strategy toward ultradispersed TiO_2_ nanocrystals on graphene. HRTEM image (right) showing nanosized TiO_2_ with highly exposed edges stacked on graphene nanosheets. Reproduced with permission.^153^ Copyright 2013, American Chemical Society. b) Schematic processes for synthesizing the hybrid of graphene and phosphorized SnO_2_. Reproduced with permission.^160^ Copyright 2018, The Royal Society of Chemistry.

With low cost, environmental friendliness and wide bandgap in UV/visible spectral region, TiO_2_ is an ideal material to hybridize with graphene for reducing charge recombination and enhancing photocatalytic and electrical performance. In Zhao's work, a simple sol–gel strategy was developed to synthesize ultradispersed TiO_2_ nanoparticles on the surface of graphene (**Figure** **11**a). As a result, an unprecedented degree of control was achieved by precisely manipulating the nucleation, growth, anchoring and crystallization of TiO_2_ during the chemical reduction of GO.^153^ The obtained hybrids showed a large surface area of ≈229 m^2^ g^−1^ and exhibited a high specific capacity of ≈94 mA h g^−1^ at 59 C, twice as that of mixed composite. Similarly, a dyade‐like graphene@TiO_2_ hybrids were produced for photocatalytic degradation of organic compounds under UV or visible irradiation.^154^ In one recent work, TiO_2_ nanoparticles and nanowires were individually hybridized onto RGO nanosheets via a hydrothermal process.^155^ In comparison, the TiO_2_ nanowires had a less agglomeration and were uniformly dispersed on graphene for more direct contact with graphene, which further improved separation and transportation of electron–hole pairs.

ZnO nanoparticles have been extensively studied as well for being hybridized with graphene. In Zhu's work, ZnO nanoparticles were first synthesized and hybridized with a certain amount of GO, and then the GO was reduced in situ to fabricate ZnO/graphene composites.^156^ Owing to the electronic interaction between ZnO and graphene, photoinduced electrons had a high migration efficiency and the recombination of charge carriers was effectively inhibited to enhance the photocatalytic activity by ≈4 times compared with pristine ZnO. Alternatively, ZnO/graphene hybrid was produced by a one‐step hydrothermal approach and showed excellent photocatalytic activity in photodegradation of deoxynivalenol under UV irradiation.^157^, ^158^ Within 30 min, 99% of deoxynivalenol (15 ppm) was photodegraded through the quick transfer of excited electrons between the conduction band of ZnO and graphene, which was 3.1 times higher than that of pure ZnO.^157^ Similarly, ZnO_1−_ *_x_*/graphene composites were synthesized to show an enhanced photocatalytic capability for degrading methylene blue.^159^ Under visible and UV light, the photocatalytic activity and photocurrent intensity of ZnO_1−_ *_x_*/graphene were greatly increased compared to that of ZnO_1−_ *_x_*. Except for TiO_2_ and ZnO, other metal oxides and hydroxide have also been hybridized with graphene for their strong synergistic activity. For example, a partially phosphorized SnO_2_/graphene composite was synthesized facilely as an attractive anode in lithium‐ion batteries through a combined hydrothermal and low‐temperature phosphorization process (Figure 11b).^160^ A superior pseudocapacitive performance was achieved by tightly anchoring a continuous mesoporous FeOOH film on graphene to form FeOOH/graphene composite.^161^

### 4.2 Hybridization of C_3_N_4_ Nanosheets

Structurally, graphitic carbon nitride (g‐C_3_N_4_) monolayer is conceived to be formed by substituting three carbon atoms of benzene ring with nitrogen atoms and then linking together via shared edges and pendant nitrogen atoms. After thermally conjugated with graphitic carbon ring, a similar in‐plane heterostructure of g‐C_3_N_4_ was prepared to expedite the separation of electron–hole pairs and promote the transport of photoelectrons, which can synergistically elongate the diffusion length and lifetime of photocarriers by 10 times relative to those achieved with pristine g‐C_3_N_4_.^162^ Besides the in‐plane polymerization, more research works reported the heterogeneous hybridization of functional molecules or nanomaterials on g‐C_3_N_4_ nanosheets due to the ease of modulation and abundant sources. Here, we summarize important advances in this field with highlighting on novel hybridization strategies and resulting functions (Table 1).

#### 4.2.1 Hybridization with Protons and Molecules

g‐C_3_N_4_ nanosheets were conveniently protonated by sonication‐driven exfoliation of g‐C_3_N_4_ in 10 m HCl solution.^163^ The resulting positively charged nanosheets were able to interact with negatively charged heparin for quenching their fluorescence, and thus employed to establish a heparin sensing platform with a detection limit of 18 ng mL^−1^. Similarly, Dong's group exfoliated g‐C_3_N_4_ nanosheets in 98 wt% H_2_SO_4_ solution and their amphoteric properties arose from the presence of both carboxyl and amino groups, suggesting an effective method to create heterostructures via an electrostatic re‐assembly of the g‐C_3_N_4_ nanosheets with charged guests such as CdS and BiOBr nanoparticles.^164^ Although this approach is impressive for functionalizing g‐C_3_N_4_ nanosheets, the use of highly concentrated acidic solution inevitably brings certain safety concerns. Recently, bulk g‐C_3_N_4_ were simultaneously exfoliated and noncovalently functionalized with 1‐pyrenebutyrate via π–π stacking interaction during mechanical grinding.^165^ With the retained optoelectronic properties, the functionalized g‐C_3_N_4_ nanosheets endowed a friendly interface for further immobilizing biomolecules or covalently linking DNA to build electro‐chemiluminescent biosensors.

#### 4.2.2 Hybridization with Nanostructures

Through developing different approaches, various as‐synthesized nanostructures were directly hybridized on g‐C_3_N_4_ nanosheets for synergistic properties. To improve photocurrent, g‐C_3_N_4_ nanosheets were hydrothermally hybridized with CdS:Mn quantum dots on glassy carbon electrode, and then used as signal generation tags in photo‐electrochemically immunosensing prostate specific antibody in biological fluids.^166^ To achieve efficient hydrogen evolution, g‐C_3_N_4_ nanosheets were functionalized with black phosphorus dots via a conventional sonication approach.^167^ The formed phosphorus–carbon bonds resulted in an effective interfacial charge separation between the two components to exhibit a high HER rate at 271 µmol h^−1^ g^−1^, which is 5.6 and 4.2 times greater compared with pristine g‐C_3_N_4_ nanosheets and black phosphorus dots, respectively. Through a self‐assembly process, Ni_2_P nanoparticles were anchored on g‐C_3_N_4_ sheets during the exfoliation of g‐C_3_N_4_ bulk under sonication to display a superior photoactivity with a hydrogen production rate of 474.7 µmol g^−1^ h^−1^.^168^ In Song's work, g‐C_3_N_4_ nanosheets were obtained through thermal exploitation of g‐C_3_N_4_ bulk followed by loading with Ag_2_CO_3_ nanoparticles to show excellent photocatalytic activity due to an increased specific surface area and enhanced charge separation rate.^169^

Similarly, Co_2_P nanorods were introduced in porous g‐C_3_N_4_ nanosheets under ultrasonication to form 1D/2D heterojunction hybrids with improved visible‐light photocatalytic capability for hydrogen generation at a rate of 53.3 µmol g^−1^ h^−1^.^170^ Meanwhile, BiPO_4_ nanorods were immobilized at the surface of g‐C_3_N_4_ nanosheets through a strong electrostatic interaction to effectively degrade Rhodamine B (94.3% within 6 min under UV light irradiation), which was ≈4.2 times and ≈1.5 times higher than that of BiPO_4_ and g‐C_3_N_4_, respectively.^171^ In addition, Bi_4_Ti_3_O_12_ nanoparticles were hybridized on g‐C_3_N_4_ nanosheets by a simple mixing–calcining process to form p–n junction heterostructures, which provided an effective separation for photogenerated electron–hole pairs and thus exhibited a higher photocatalytic ability than bare Bi_4_Ti_3_O_12_ and g‐C_3_N_4_.^172^ Further, the (001)‐exposed anatase TiO_2_ nanosheets were combined with g‐C_3_N_4_ via a solvent evaporation process for improving the photocatalytic reactivity in degrading organic molecules under the irradiation of UV and visible light.^173^ In addition, negatively charged g‐C_3_N_4_ was electrostatically hybridized with positively charged NiAl‐layered double hydroxides via a strong electrostatic interaction to achieve remarkable performance in photocatalytic reduction of CO_2_ and production of H_2_ under the irradiation of visible light.^174^

**Figure 12:**
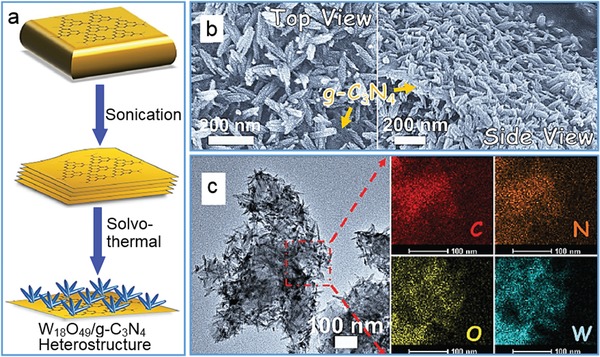
The growth of W_18_O_49_ nanograsses on g‐C_3_N_4_ nanosheets. a) Synthetic approach to W_18_O_49_/g‐C_3_N_4_ heterostructure. b) SEM images of top and side view of the heterostructure. c) TEM image and the corresponding elemental mapping images of the heterostructure. Reproduced with permission.^175^ Copyright 2017, Wiley‐VCH.

Through optimizing reaction conditions, various functional nanostructures were heterogeneously grown on g‐C_3_N_4_ nanosheets to achieve synergistic functions effectively. Dong's group demonstrated the growth of W_18_O_49_ nanograsses on g‐C_3_N_4_ nanosheets for producing a nonmetal plasmonic Z‐scheme photocatalyst (**Figure** **12**).^175^ The g‐C_3_N_4_/W_18_O_49_ hybrids harvested photon energies spanning from UV to near‐infrared spectral region and possessed improved charge‐carrier dynamics for boosting the generation of long‐lived active electrons during photocatalytic reduction of protons into H_2_. Similarly, g‐C_3_N_4_/anatase TiO_2_ hybrids were hydrothermally synthesized through heterogeneous growth process, and the exposed TiO_2_ (001) facets in heterojunction ensured the efficient separation of photogenerated carriers and accordingly enhanced the photocatalytic evolution of hydrogen.^176^ In addition, ultrathin g‐C_3_N_4_/BiOCl heterostructure nanosheets were successfully prepared by a modified solvothermal method to exhibit an excellent photocatalytic performance.^177^ Under the irradiation of visible light, 95% of 4‐chlorophenol was removed within 2 h by using the oxygen vacancy‐rich hybrids, which was ≈12.5 and 5.3 times greater than that of pure BiOCl and g‐C_3_N_4_, respectively.

At the same time, functional nanostructures were also synthesized in situ on g‐C_3_N_4_ nanosheets for highly synergistic properties. In Wang's work, the fused g‐C_3_N_4_/TiO_2_ heterostructures were obtained through high temperature production of g‐C_3_N_4_ in mesoporous TiO_2_ spheres pre‐infiltrated with molten dicyandiamide, and their strong interfacial connection enhanced the photocatalytic activity due to the promoted electron transfer.^178^ Alternatively, Hao et al. directly synthesized macro/mesoporous g‐C_3_N_4_/TiO_2_ heterostructures by a facile calcination approach using tetrabutyl titanate and melamine as feedstocks, in which the added content of melamine greatly influenced on their photocatalytic activity.^179^, ^180^ At an optimal loading content, the photocatalytic performance was 18.7 and 3.5 times better than that of pure TiO_2_ and g‐C_3_N_4_, respectively.^179^ For the degradation of Rhodamine B, the reaction rate constant was up to 47.8 × 10^−3^ min^−1^, 7.2 and 3.1 times higher than pure TiO_2_ and g‐C_3_N_4_, respectively.^180^

In addition, ternary nanohybrids were further produced in recent years. To improve the performance in electrochemical applications, g‐C_3_N_4_/ZnO heterostructures were hybridized with other nanostructures to fabricate ternary composites such as g‐C_3_N_4_/ZnO/GO and g‐C_3_N_4_/ZnO/polyaniline.^181^, ^182^ The loading of GO increased the absorption intensity in visible region and charge separation efficiency was greatly improved to enhance photocatalytic activity for two times.^181^ For g‐C_3_N_4_/PANI/ZnO composites, charge separation efficiency, specific surface area and visible light harvesting were simultaneously improved for achieving superior visible photocatalytic capability in degradation of methylene blue and 4‐chlorophenol, which was ≈3.6 and ≈3.3 times higher compared to those for g‐C_3_N_4_, respectively.^182^ Recently, well‐aligned ZnO nanorods were synthesized on g‐C_3_N_4_ nanosheets via a microwave‐assisted hydrothermal treatment due to strong Zn–N interaction, and then deposited with Pt nanoparticles to produce g‐C_3_N_4_/ZnO/Pt composites with excellent sensitivity/selectivity and rapid response/recovery rate for detection of ethanol and NO_2_.^183^ Another interesting work was to deposit CoO*_x_* nanoparticles on WO_3_/g‐C_3_N_4_ heterostructures for creating ternary WO_3_/g‐C_3_N_4_/CoO*_x_* composites with significantly enhanced photo‐electrochemical water oxidation.^184^

### 4.3 Hybridization of Black Phosphorus Nanosheets

With high charge‐carrier mobility and thickness‐dependent direct‐bandgap in mid‐infrared regime, black phosphorus (BP) nanosheets have become a newly emerging class of 2D nanomaterials since their first fabrication in 2014.^185^ Their further combination with a range of organic molecules and inorganic nanomaterials offers more versatile and robust hybrids or composites for various outstanding performances in solar fuel production and environmental remediation.^186^, ^187^ In this part, recent achievements on BP‐based hybrids are summarized with a focus on their improved stability and enhanced applications in energy conversion and catalysis (Table 1).

#### 4.3.1 Improved Passivation/Stabilization

**Figure 13:**
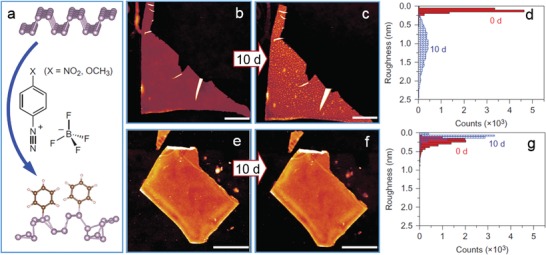
Chemical passivation of BP layers via covalent modification. a) Reaction scheme of benzene‐diazonium tetrafluoroborate derivatives on BP layer. b,c) AFM characterization and d) histograms of the surface roughness of pristine BP flake immediately after exfoliation and after ten days of ambient exposure. e,f) AFM characterization and g) histograms of the surface roughness of the BP morphology immediately after functionalization with 4‐nitrobenzene‐diazonium (4‐NBD) for 30 min and after ambient exposure for ten days. Scale bars are 2 µm. Reproduced with permission.^190^ Copyright 2016, Nature Publishing Group.

As reported, bulk crystal of BP is stable under ambient conditions for at least a few months, but single‐ and few‐layer BP nanosheets are rapidly degraded into oxidized phosphorus species in the presence of moisture and oxygen (complete degradation within several hours).^188^ The degradation is resulted from the formation of P*_x_*O*_y_* via the easy‐going reaction of lone pair electrons of BP with oxygen, which suggests an effective strategy for mitigating oxidation of BP by occupying the lone pair electrons with other elements.^189^ To this end, various effective approaches were developed for protecting BP nanosheets by surface modification with organic molecules. An very successful example was reported by Hersam's group,^190^ in which covalent functionalization of aryl diazonium effectively suppressed the chemical degradation of BP nanosheets upon ambient exposure for three weeks (**Figure** **13**). Meanwhile, this chemical modification spontaneously formed phosphorus–carbon bonds to optimize the electronic features of BP nanosheets, ultimately leading to a strong and controlled p‐type doping to improve FET mobility and on/off current ratio. In Yang's work, BP nanosheets were stabilized via edge‐selective functionalization with hydrophobic C_60_ molecules, which served as a sacrificial shield to protect BP sheets from degradation and also promoted the photoinduced electron transfer to achieve excellent photo‐electrochemical and photocatalytic performances.^191^

Besides covalent modification, noncovalent functionalization on BP nanosheets is also effective for stabilizing BP nanosheets under ambient conditions. Typically, Hirsch's group modified BP nanosheets with perylene diimide via van der Waals interaction, leading to a remarkable stabilization of BP flakes against oxidized degradation.^192^ Similarly, surface coordination of BP nanosheets by titanium sulfonate was also reported to obtain high stability in air and water.^193^ Recently, AlO*_x_* overlayers were found to effectively inhibit degradation of BP nanosheets in ambient conditions, endowing the encapsulated BP FETs with high on/off ratio (≈10^3^) and good mobility (≈100 cm^2^ V^−1^ s^−1^) for at least 2 weeks.^194^

#### 4.3.2 Synergistically Enhanced Applications

The transport behavior of BP nanosheets is sensitive to other species on their surface such as adatoms, adsorbates or dopants, which not only improve their stability, but also regulate their property for better applications. Representatively, Cu adatoms on BP nanosheets provided electron doping properties to lower the threshold voltage of nanosheets without reducing transport properties.^195^ Further, 7,7,8,8‐tetracyano‐*p*‐quinodimethane on BP nanosheets offered electron‐withdrawing property to render electron transfer from BP to organic moiety,^193^ leading to a low on‐state resistance (3.2 Ω mm), and thus high field‐effect mobility (229 cm^2^ V^−1^ s^−1^) and drain current (532 mA mm^−1^).^196^ In addition, the Se‐doped BP nanosheets exhibited reliable electrical features at ambient environment including high on/off current ratios at 10^5^ and mobility at 561 cm^2^ V^−1^ s^−1^.^197^ When applied into 2D photodetectors, they greatly improved photoelectrical properties with remarkably increased responsivity from 0.765 to 15.33 A W^−1^, i.e., 20‐fold enhancement compared to pristine BP.

Metal and semiconductor nanocrystals were also hybridized on BP nanosheets for various enhanced applications. For instance, Ag nanoparticles were modified on BP nanosheets via covalent linkage at their interface and Ag–Ag interaction during chemical reduction of AgNO_3_, resulting in plasmonic hybrids with a significant rise in photoactivity for visible light photodegradation of Rhodamine B, with an enhancement up to ≈20‐fold compared to pristine BP nanosheets.^198^ Similarly, BP@TiO_2_ hybrid photocatalysts were synthesized to offer enhanced photocatalytic performance and maintain ≈92% photoactivity activity after 15 runs.^199^ In addition, SrTiO_3_ was also hybridized on BP nanosheets to achieve the giant photoresponsivity with a photoinduced current change of more than 10^5^ A W^−1^.^200^

**Figure 14:**
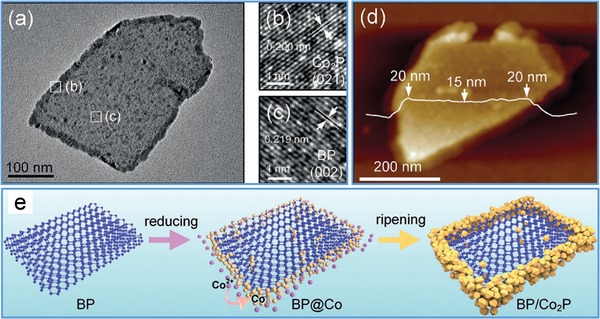
The selective growth of Co_2_P nanocrystals on the BP edges. a) TEM image of one BP/Co_2_P nanosheet. b) HR‐TEM image of Co_2_P corresponding to the edge location and c) HR‐TEM image of BP corresponding to the center position. d) AFM image and line‐scan of one BP/Co_2_P nanosheet. e) Schematic synthesis of BP/Co_2_P heterostructures. Reproduced with permission.^205^ Copyright 2018, Wiley‐VCH.

Recently, more BP hybrids were fabricated by using other functional nanomaterials.^201^, ^202^, ^203^, ^204^ For example, strong electron doping of BP with Cs_2_CO_3_ nanoparticles significantly enhanced the electron mobility to ≈27 cm^2^ V^−1^ s^−1^, while surface decoration of BP with MoO_3_ nanoparticles demonstrated a giant hole‐doping effect.^201^ The Zn_0.5_Cd_0.5_S nanoparticles were hybridized onto the pre‐produced BP nanosheets via an ultrasonic process to achieve the hydrogen production rate as high as 137.17 mmol g^−1^ h^−1^ under the irradiation of visible light, 5 times higher than that for the pristine Zn_0.5_Cd_0.5_S.^202^ After enhancing the visible‐light photocatalytic activity by using synthesized Z‐scheme photocatalytic BP/BiVO_4_ heterostructures,^204^ the hydrogen and oxygen production rates were increased to ≈160 and ≈102 mmol g^−1^ h^−1^ under the irradiation of light with a wavelength longer than 420 nm.^203^ Co_2_P nanocrystals were selectively created at the reactive BP edges via a solvothermal reaction to produce in‐plane BP/Co_2_P heterostructures with better and more stable electrocatalytic activities for hydrogen and oxygen evolution due to the improved conductivity and more active sites (**Figure** **14**).^205^ Alternatively, a ternary composite of BP, Ketjenblack and carbon nanotubes was fabricated through high‐energy ball milling and further used as a anode material in sodium‐ion batteries to exhibit a very high initial Coulombic efficiency (>90%) and excellent cyclability at high rate of charge/discharge.^206^

Another important application of BP‐based hybrids is to fabricate p–n heterojunction by combing p‐type BP and n‐type nanomaterials. In Lee's work, BP and MoS_2_ nanoparticles were hybridized into a photodetector with enhanced photoresponsivity and detectivity, and their performance was dependent on the density of MoS_2_ nanoparticles on BP layer (e.g., the response time was reduced with the increased density).^207^ Similarly, BP nanosheets were facilely hybridized with an underlying n‐doped GaAs substrate to form a p–n heterojunction diode, which exhibited close‐to‐ideal diode behavior at low bias, e.g., its photoresponse was evenly distributed on the entire junction area and an external quantum efficiency of 10% was achieved at zero bias.^208^ In addition, the hybridization of BP nanosheets and ZnO nanowires was also utilized to construct the p–n heterojunction diode and BP‐gated junction FETs on glass.^209^ With the van der Waals junction interface between BP and ZnO, the p–n diode displayed a high on/off ratio of ≈10^4^ in static rectification and showed kilohertz dynamic rectification as well.

### 4.4 Hybridization of Metal Oxide Nanosheets

Owing to natural abundance, environmental friendliness, low cost, and wide bandgap across visible to ultraviolet region, transition metal oxide (TMO) nanosheets have recently attracted a remarkable attention as electrode layers in rechargeable batteries or as light‐harvesting agents for solar fuel production and environmental remediation. As demonstrated, conductive heterogeneous components such as carbon nanotubes and graphene are hybridized on metal oxide nanosheets to significantly overcome their drawback of low conductivity.^210^ In addition, the introduction of other functional materials on various metal oxide nanosheets also facilitates their utilization in diverse areas. Here we overview the recent developments on the modification and hybridization of metal tri, di and monoxide nanosheets with various functional species as classified based on the oxygen content in metal oxides.

#### 4.4.1 Metal Trioxide Nanosheets

To give rise to strong localized surface Plasmon resonance in visible and near‐infrared spectral region, oxygen vacancies were introduced in MoO_3_ to produce nonstoichiometric MoO_3−_ *_x_* sheets in the presence of ascorbic acid. This was because hydrogen ions were intercalated in the lattice of MoO_3_ and bonded with oxygen atoms to form water molecules and meanwhile the electron of ascorbic acid was transferred to reduce Mo^6+^.^211^ Further, Pd tetrahedrons were synthesized on MoO_3−_ *_x_* surfaces through a one‐pot wet‐chemical approach.^212^ The electron‐donating feature of oxygen vacancies regulated electronic structure of Pd nanostructures for improving the catalytic activity of Pd/MoO_3−_ *_x_* hybrids, and thus an excellent performance was achieved for selective hydrogenation of α,β‐unsaturated aldehydes to its saturated aldehydes with high conversion (97%) and selectivity (96%). In Ma's work, the epitaxial growth of Au nanocrystals on MoO_3_ nanosheets was realized under ultraviolet irradiation by using MoO_3_ nanosheets as both electron carriers and sacrificial templates, which were used as homogeneous SERS substrates for constructing a detection platform toward malachite green pigment.^213^ Similarly, 1–3 nm‐sized Pt nanoparticles were synthesized on MoO_3_ nanosheets with an uniform dispersive density, and the Pt‐MoO_3_ composite displayed an increased peroxidase‐like catalytic activity superior to the MoO_3_ nanosheets, Pt nanoparticles and their physical mixture.^214^ Recently, the growth of α‐Fe_2_O_3_ nanorods on surface of α‐MoO_3_ nanobelts was also realized by a hydrothermal approach for developing xylene gas sensor with high response and low operating temperature.^215^

**Figure 15:**
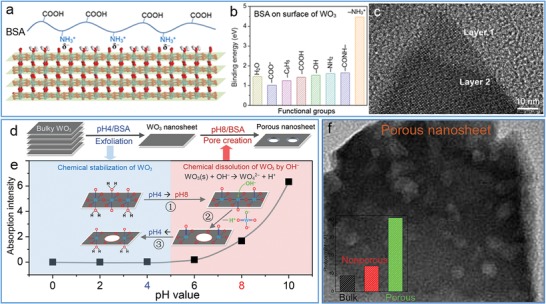
Controlled exfoliation of WO_3_ nanosheets in a pH 4 solution and subsequent hole creation on resulting nanosheets in a pH 8 solution. a) Schematic illustration for the electrostatic binding of BSA on WO_3_ surface. b) Calculated binding energies. c) High‐resolution TEM images of WO_3_ nanosheets. Reproduced with permission.^42^ Copyright 2017, Wiley‐VCH. d) Schematic illustration from the WO_3_ bulk to WO_3_ nanosheets to porous nanosheets. e) pH‐dependent evolution of the absorption intensity of WO_4_^2−^ ions after WO_3_ powder is dissolved in aqueous solutions at different pH values for 24 h. f) TEM image of porous WO_3_ nanosheets and the comparison on catalytic ability of different samples (inset). Reproduced with permission.^216^ Copyright 2018, American Chemical Society.

In our previous work, the electrostatic interaction between BSA and WO_3_ was facilely used to directly exfoliate monoclinic WO_3_ bulk into nanosheets (**Figure** **15**a–c).^42^ The capping of BSA on surface of WO_3_ nanosheets suggested a chemical dissolution process to create hole on WO_3_ sheets.^216^ As illustrated in Figure 15d,e, through surface blocking by BSA, WO_3_ nanosheets were treated in pH 8 solution to readily form holes due to the reaction of WO_3_ with OH^−^ ions, which significantly increased the bandgaps of WO_3_ and provided more edge active sites. Compared to WO_3_ bulk and nonporous sheets, the holey WO_3_ sheets exhibited much higher photocurrents and better performance in selective adsorption of herbicide and photocatalytic degradation of crystal violet (Figure 15f). In Mul's work, the photodeposition of Pt nanocrystals on WO_3_ plates was achieved through the preferential adsorption of [PtCl_6_]^2−^ ions on the positively charged facets/edges resulted from the non‐uniform distribution of intrinsic charges at surface of WO_3_ plates in an aqueous solution.^217^

#### 4.4.2 Metal Dioxide Nanosheets

**Figure 16:**
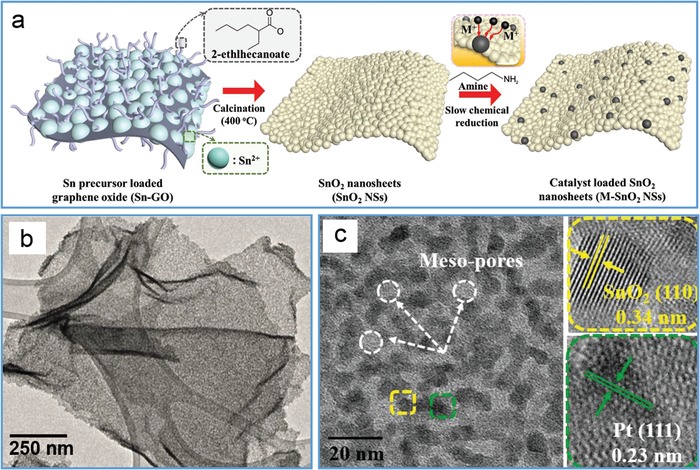
Chemical synthesis of Pt‐SnO_2_ nanosheets. a) Schematic illustration of synthetic process. b) TEM image of SnO_2_ nanosheets functionalized by Pt nanoparticles. c) High resolution TEM image of Pt‐SnO_2_ nanosheets. Reproduced with permission.^219^ Copyright 2018, Wiley‐VCH.

By an impregnation method, Pd nanocrystals were facilely grown on δ‐MnO_2_ nanosheets to form a promising hybrid catalyst for Li–O_2_ cells,^218^ which exhibited various outstanding performance (e.g., low polarization, good rate capability and long cycle life) owing to the synergetic catalytic effect via the charge transfer between δ‐MnO_2_ and Pd. At a high current density of 1600 mA g^−1^, the rate capability was 2400 mA h g^−1^ while terminal charge/discharge voltages were as low as 4.2 V/2.58 V. In addition, Pt‐SnO_2_ nanosheets with high mechanical flexibility were prepared via the coating of Sn precursor on GO nanosheets and subsequent calcination at 400 °C to form SnO_2_ nanosheets, followed by functionalization with Pt (**Figure** **16**).^219^ The hybrid sheets were further assembled on a highly stable film heater to construct an ultrasensitive flexible sensing platform for dimethyl sulfide. In Wen's work, ultrafine Co_3_O_4_ nanocrystals were decorated on atomic‐thick TiO_2_ nanosheets via hydrothermal approach and the hybrid was utilized as cathode catalytic materials in Li–O_2_ batteries.^220^ The Co doping in the hybrids induced a great amount of oxygen vacancies in TiO_2_ sheets, and thus led to high specific capacity, good cycling stability, and low polarization.

#### 4.4.3 Metal Monoxide Nanosheets

Recently, superior high power density/energy density and durability were achieved in zinc–air batteries by multiscale structural engineering on Ni‐doped CoO sheets.^221^ The doping of Ni greatly increased the reaction activity in intrinsic oxygen reduction and thus produced a favorable surface for oxygen diffusion with a high electrocatalytic active surface area. As a result, the primary zinc–air battery showed various outstanding performances including a high discharge peak power density at 377 mW cm^−2^, working stably for >400 h at 5 mA cm^−2^ and a small charge–discharge voltage of 0.63 V, outperformed the battery composed of the state‐of‐the‐art Pt/C catalyst. As a result, 17 light‐emitting diodes can be powered and an iPhone 7 mobile phone can be charged at laboratory level by using all‐solid‐state coin cells assembled by using Ni‐doped CoO nanosheets.

#### 4.4.4 Bimetallic Oxide Nanosheets

Metal–organic framework (MOF)‐templated method was employed to prepare porous bimetallic Co_3_O_4_/ZnO nanosheets via a one‐pot room‐temperature reaction of Co(NO_3_)_2_, Zn(NO_3_)_2_ and 2‐methylimidazole in water, providing more active sites from oxygen vacancies for electrochemical reaction in both lithium‐ion and sodium‐ion batteries.^222^ In another work, porous ZrO_2_–SiO_2_ sheets were first prepared by using GO as a template and subsequently impregnated with a tungsten precursor to form ultrasmall WO_3_ nanoparticles via calcination at 500 °C.^223^ Additionally, an asymmetric supercapacitor was fabricated by using self‐assembled NiCo_2_O_4_/MnO_2_ and surface‐polymerized MoO_3_@PPy as the positive and negative electrode in Na_2_SO_4_ electrolyte,^224^ which exhibited overall areal capacitance retentions of 97.5% and 86.2% after 6000 cycles, respectively.

### 4.5 Hybridization on Other Types of Nanosheets

Layered metal hydroxides have been used as host components to fabricate hybrid systems for improving their catalytic performance via synergistic enhancements originated from the hybrid interfaces.^225^ In this part, we introduce the recent hybridization works for better understanding and accelerating the developments of varied advanced hybrids. For example, CuSe nanosheets were vertically aligned on Ni(OH)_2_ sheets to fabricate porous frameworks, which facilely resolved the poor conductivity and restacking problems of Ni(OH)_2_ nanosheets. Moreover, a 3D electrons or ions transport pathway was simultaneously created to exhibit high volumetric specific capacitance (38.9 F cm^−3^), good cycling performance and superb flexibility/mechanical stability.^226^ In Ho's work, MoS_2_ nanobelts were interfaced on porous Ni(OH)_2_ nanosheets to form 2D‐on‐2D hierarchical architectures with impressive capabilities for complementary energy storage and energy conservation, which were further employed for delivering supercapacitive charge storage and electrochromic optical modulation to display bifunctional supercapacitive–electrochromism properties.^227^ Recently, ultrathin nanosheets of multiple‐component metal hydroxide were synthesized via the progressive decomposition of metal–boron nanocrystals (metals = Fe, Co, and Ni) to release boron species together with oxidized metal.^228^ The hybrid nanosheets with high specific surface area exhibited an superb catalytic activity in the Heck reaction. In addition, the hybridization of CoO nanocrystals on CoNi layered double hydroxide was also achieved by in situ reduction and interface‐directed assembly in air, in which the interfacial tension drove the strong extrusion of hydrated metal–oxide clusters for the hybridization.^229^

Other types of ultrathin nanosheets were also used as supports to develop hybrids for better applications. For instance, Dai's group hybridized Co‐doped FeS_2_ sheets with carbon nanotubes to fabricate a composite catalyst with high activity and stability for HER in acidic solutions, exhibiting a low overpotential of ≈0.12 V at 20 mA cm^−2^, a small Tafel slope of ≈46 mV decade^−1^ and a long‐term durability over 40 h of reaction.^230^ It was found that the catalytic activity was dependent on the heteroatomic interactions between the two moieties, and DFT results revealed that the high activity after Co doping was attributed to a significant reduction in the kinetic energy barrier of hydrogen atom adsorption on FeS_2_ surface. Zhang's group demonstrated the high‐yield production of ultrathin Ti*_x_*Ta_1−_ *_x_*S*_y_*O*_z_* sheets by a solution‐processed method using Ti*_x_*Ta_1−_ *_x_*S_2_ as precursor, which showed a strong near‐infrared absorbance at 808 nm with a extinction coefficient of 54.1 Lg^−1^ cm^−1^ and a high photothermal conversion efficiency as high as 39.2%.^231^ Alternatively, Huang's group reported in situ hybridization of cobalt 1,4‐benzenedicarboxylate on Ti_3_C_2_T*_x_* sheets through an interdiffusion reaction‐assisted method to exhibit an excellent performance when applied in oxygen evolution reaction, outperformed those by using IrO_2_‐based catalyst.^232^

## 5 Interspecies Hybridization of Different 2D Nanomaterials

When stacking different 2D nanomaterials with diverse physical properties into heterostructures, charge redistribution might occur between neighboring nanosheets and thus induce structural changes in each other to generate numerous exciting physical phenomena. The extended range of synergistic properties provide a promising potential for utilizing such heterostructures as functional materials in future electronic and optoelectronic devices (Table 1). For instance, the highest‐mobility graphene transistors were made by encapsulating graphene with hexagonal boron nitride (BN) sheets.^30^ In the past decade, the interspecies hybridizations of different 2D nanomaterials were mainly performed by interlayer vertical stacking via van der Waals force or in‐plane concatenation via covalent bonds, which were realized through mechanical assembly/selective growth of one nanosheet on another one or lateral epitaxial growth, respectively. In this section, we comprehensively overview the interspecies hybridization of different 2D materials, emphasizing their design concept, fabrication mechanism, and synergetic effects/improved performance in heterostructured devices.

### 5.1 Hybridization of TMD Nanosheets with Others

Various TMD nanosheets have been obtained by adjusting the types of transition metals and chalcogens in TMDs. With the diversity of TMD nanosheets, we first describe the synergistic hybridization between different TMD nanosheets and then summarize the systematic combination of TMD nanosheets with other 2D materials.

#### 5.1.1 Two Types of TMD Nanosheets

*Physical Stacking*: In Rajamathi's work, the as‐ammoniated MoS_2_ and WS_2_ nanosheets were exfoliated and randomly restacked to form hybrids in their mixed dispersion during evaporating its polar solvent.^233^ Alternatively, the successive mechanical exfoliations were rationally employed for precisely controlling the stacked structure to fabricate the heterostructures of different TMD nanosheets. For instance, Wilson et al. synthesized the MoSe_2_/WSe_2_ heterobilayers with a high binding energy of >200 meV between interlayer excitons, an order of magnitude higher than that in layered GaAs materials.^234^ From the band structure across the heterojunction, this hybridization significantly modified their bands at Γ, while the edge of valence band remained at K point. Moreover, the weak hybridization of bands in the K‐point valleys yielded a valence band offset of 300 meV to indicate type II band alignment.

**Figure 17:**
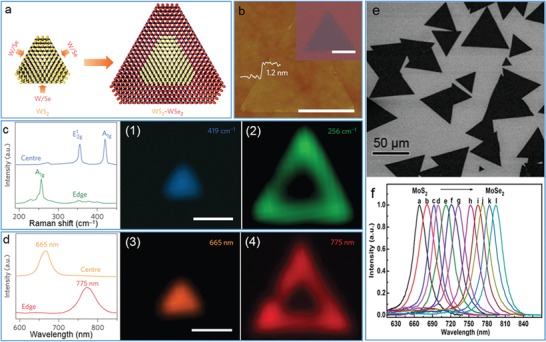
Hybridization of different TMD nanosheets. a) Schematic of lateral epitaxial growth of WS_2_–WSe_2_ heterostructures. b) AFM image of WS_2_–WSe_2_ lateral heterostructure. Inset is optical image of a triangular domain. Scale bars = 5 µm. c) Raman and d) photoluminescence characterization in the center region and the peripheral region of WS_2_–WSe_2_ heterostructures: 1,2) are Raman mapping at 419 and 256 cm^−1^, respectively; 3,4) are photoluminescence mapping at 665 and 775 nm, respectively. Reproduced with permission.^236^ Copyright 2014, Nature Publishing Group. e) SEM image of the ternary MoS_2_ *_x_*Se_2(1−_ *_x_*_)_ nanosheets (*x* = 0.48). f) Normalized photoluminescence spectra of the MoS_2_ *_x_*Se_2(1−_ *_x_*_)_ nanosheets excited with a 488 nm argon ion laser. Curves (a–l) are the MoS_2_ *_x_*Se_2(1−_ *_x_*_)_ nanosheets synthesized at temperature of 830 to 796 °C, respectively. Reproduced with permission.^238^ Copyright 2014, American Chemical Society.

*Epitaxial Growth*: The 2D nature together with small lattice mismatch between different TMD nanosheets renders them ideal substrates to construct vertical and lateral heterostructures at atomic scale by means of CVD‐based epitaxial growth, exhibiting the potential applications in electronic and optoelectronic devices.^235^ Duan et al. fabricated compositionally controlled WS_2_–WSe_2_ or MoS_2_–MoSe_2_ lateral heterojunctions via in situ CVD deposition (**Figure** **17**a,b).^236^ Experimentally, a triangular WS_2_ sheet was deposited on a silicon oxide substrate, and its peripheral edges with unsaturated dangling bonds further served as the active growth front for extending it in lateral direction to form WS_2_–WSe_2_ heterostructures. Raman and photoluminescence mapping exhibited its clear structural and optical modulation (Figure 17c,d), and electrical transport studies showed lateral p–n diodes and photodiodes in the WSe_2_–WS_2_ hybrid nanosheets.

*Alloying of TMD Nanosheets*: Besides the hybridization of different TMD nanosheets, the direct growth of alloyed nanosheets is another approach to produce hybrids. In Suenaga's work, a series of mixed Mo_1‒_ *_x_*W*_x_*S_2_ single layers (*x* = 0, 0.2, 0.5, 0.8, and 1) was fabricated through chemical‐vapor transport of starting materials while the alloying degree of metal elements was quantified by atomically resolved scanning transmission electron microscope.^237^ Alternatively, Duan's group reported a one‐step CVD strategy to simultaneously grow alloyed MoS_2_ *_x_*Se_2(1−_ *_x_*_)_ triangular sheets with complete composition tunability (Figure 17e).^238^ Both Raman and photoluminescence observations showed the consistence of tunable optical features with the composition of alloyed sheets. More interestingly, all samples exhibited a single band edge emission peak from 668 to 795 nm (Figure 17f), which brought about an exciting chance for manipulating the fundamental physical characteristics of 2D nanomaterials. Meanwhile, the alloyed nanosheets were also obtained via exfoliating their parent bulk crystals (e.g., Ta_2_NiS_5_ and Ta_2_NiSe_5_) for high‐yield and scalable production of ultrathin ternary chalcogenide sheets in solution by using an electrochemical Li‐intercalation method.^239^ With a production yield at ≈86%, Ta_2_NiS_5_ nanosheets were used to develop highly sensitive and selective fluorescent sensor for detecting DNA with an detection limit of 50 × 10^−12^ m.

#### 5.1.2 TMD Nanosheets with Graphene

Due to easy‐operating process and promising properties, TMD nanosheets have been readily hybridized with graphene or RGO in the recent years. This further provides more opportunities to develop novel devices with enhanced functionalities. Experimentally, the hybridization can be realized through simply mixing TMD nanosheets and graphene in a proper solution, followed by self‐assembly or random stacking. Also, the pre‐designed hybridization can also be achieved by rationally transferring TMD nanosheets onto graphene (vice versa) or directionally growing TMD nanosheets onto graphene.

*Self‐Assembling or Inter‐Stacking*: In Wallace's work, the mixed dispersion of MoS_2_ nanosheets and GO spontaneously self‐assembled into free‐standing MoS_2_–RGO network with a 3D porous structure after gelation and subsequent reduction.^240^ With 75 wt% MoS_2_, the resulting hybrids exhibited a high reversible capacity of 800 mA h g^−1^ at a current density of 100 mA g^−1^, and demonstrated a superb rate capability and excellent cycling stability (e.g., no capacity drop was found over 500 charge/discharge cycles at a current density of 400 mA g^−1^). Similarly, MoSe_2_/graphene heterostructures were prepared by depositing graphene and MoSe_2_ flakes on substrates via a solution processing approach, and their electrochemical coupling effectively enhanced the electrocatalytic activity in hydrogen evolution reaction, resulting in a cathodic current density of 10 mA cm^−2^ at overpotential of 100 mV and an exchange current density of 0.203 µA cm^−2^.^241^

*Transfer of TMD Nanosheets onto Graphene or Vice Versa*: With its chemical inertness together with the lack of dangling bonds, graphene is usually used as building blocks or supports to combine with other 2D materials via van der Waals interactions. Ouerghi's group transferred monolayer MoS_2_ nanosheets onto graphene to obtain a heterostructure of MoS_2_/graphene.^242^ With their rather high binding energies, graphene band structure was significantly modified to impose a superperiodic potential via the opening of several miniband gaps due to the overlay of MoS_2_ layer and graphene lattice. In Zhang's work, a flexible transistor array composed of MoS_2_/graphene was developed by using MoS_2_ nanosheets as active channel and RGO as drain and source electrodes to achieve a much higher sensitivity in a gas sensor compared to RGO nanosheets as active channel.^243^ Furthermore, the functionalization of Pt nanoparticles on MoS_2_ thin film increased the sensitivity by up to ≈3 times. Meanwhile, graphene was transferred onto MoS_2_ nanosheets to form graphene‐on‐MoS_2_ heterostructures with excellent optoelectronic functionalities such as highly sensitive photodetection and gate‐tunable persistent photoconductivity.^244^ Their responsivity was reported to be ≈1 × 10^10^ A W^−1^ at 130 K and ≈5 × 10^8^ A W^−1^ at room temperature, indicating the most sensitive graphene‐based photodetector. In another work, the unique electronic features of MoS_2_ monolayer and the high conductivity of graphene were combined to construct a 2D heterostructure with information storage capability.^245^ Owing to its bandgap and 2D structure, monolayer MoS_2_ was very sensitive to the change of charges in charge trapping layer, leading to a factor of 10^4^ difference between memory program and erase states.

**Figure 18:**
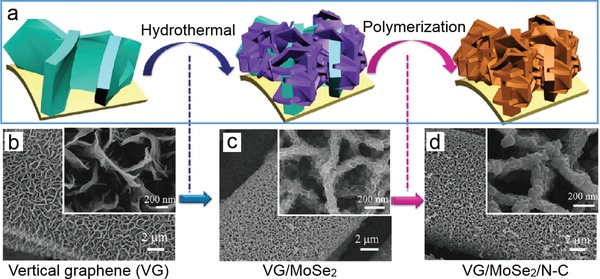
a) Schematic illustration for synthesizing sandwiched VG/MoSe_2_/N‐C core/shell arrays and b–d) SEM images of typical products. VG represents vertical graphene and N‐C represents N‐doped carbon in shell. Reproduced with permission.^250^ Copyright 2017, Wiley‐VCH.

*Growth of TMD Nanosheets on Graphene*: To achieve the selective growth of TMD nanosheets on graphene or RGO, some assistant agents are usually required in reaction solution. Typically, under the assistance of L‐cysteine, MoS_2_/graphene composites were produced through a hydrothermal method by using sodium molybdate and GO as starting materials, followed by an annealing in H_2_/N_2_ atmosphere.^246^ When the Mo:C molar ratio was set as 1:2, the hybrids served as an anode material in Li‐ion batteries to exhibit the highest specific capacity of ≈1100 mA h g^−1^ at a current of 100 mA g^−1^. Similarly, Wu's group deposited p‐type MoS_2_ nanosheets onto n‐type N‐doped RGO sheets under the assistance of L‐ascorbic acid to fabricate multiple nanoscale p–n junctions with significantly enhanced charge generation and greatly suppressed charge recombination.^247^ Alternatively, WS_2_ nanosheets were grown on RGO nanosheets via a hydrothermal reaction by using cetyltrimethylammonium bromide (CTAB) as surfactant, and CdS nanorods were further modified on the hybrids via a solvothermal method to form a ternary composite for serving as a highly active photocatalyst under visible light irradiation.^248^ Following the similar process, Zhao et al. realized the directional growth of MoSe_2_ sheets on graphene by a CTAB‐directed hydrothermal approach and the resulting 2D composite exhibited strong electronic coupling to facilitate both electron and Na^+^ ions transfer across the interface and reversible insertion/extraction for fast pseudocapacitive Na‐ion storage.^249^ In Tu's recent work, wrinkled MoSe_2_ sheets were first synthesized on vertical graphene and then coated with N‐doped carbon shell to form sandwiched core/shell arrays (**Figure** **18**), which played a positive role in enhancing electrochemical performance owing to the greatly improved conductivity and highly porous structure for fast ion diffusion.^250^

#### 5.1.3 TMD Nanosheets with g‐C_3_N_4_ Nanosheets

Owing to high surface activity, g‐C_3_N_4_ nanosheets have been regularly utilized as templates for growing TMD nanosheets on them. In Ajayan's work, 1T‐MoS_2_ was grown on oxygenated C_3_N_4_ monolayer by a solvothermal reaction, and the heterojunction between 2D nanosheets can minimize the Schottky barrier and improve the charge transfer efficiency.^251^ In Ng's work,^252^ MoS_2_ nanosheets were coupled into porous C_3_N_4_ via van der Waals interaction to create MoS_2_/C_3_N_4_ heterostructures by an ultrasonication‐assisted wet‐chemical approach. The optimized heterostructures with 0.05 wt% MoS_2_ exhibited a reaction rate constant as high as 0.301 min^−1^, 3.6 times higher than that of bare C_3_N_4_. Further, MoS_2_ as electron trapper extended the lifetime of separated electron–hole pairs, and the accumulated holes on C_3_N_4_ surface oxidized organic dye directly, a predominant reaction in the photocatalytic degradation of organic pollutants for environmental remediation.

#### 5.1.4 TMD Nanosheets with BP Nanosheets

**Figure 19:**
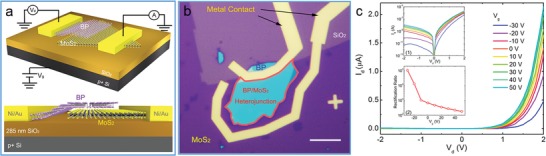
A van der Waals heterojunction p–n diode based on BP and MoS_2_ nanosheets. a) Schematic device structure. b) Optical image of device structure. The dark purple region is monolayer MoS_2_ while the blue flake is few‐layer BP. Scale bar, 10 µm. c) Gate tunable *I*–*V* characteristics of the p–n diode. The top inset shows the *I*–*V* characteristics under semilog scale. The bottom inset shows the rectification ratio as a function of back gate voltage *V*_g_. Reproduced with permission.^254^ Copyright 2014, American Chemical Society.

With complementary band structures, TMD and BP nanosheets have been often hybridized to exhibit unimaginable synergistic effects for various important applications such as p–n diodes and photoresponsivity. Hong et al. investigated the photocurrent generation at a vertical p–n heterojunction between BP and MoS_2_ flakes by polarization‐, wavelength‐, and gate‐dependent scanning photocurrent measurements.^253^ When the incident photon energy was higher than the direct bandgap of MoS_2_, the photocurrent response demonstrated a competitive behavior across the p–n junction. When the incident photon energy resided between the two bandgaps of MoS_2_ and BP, the photocurrent response exhibited the same polarization dependence as that at BP–metal heterojunctions. In another work, Ye's group reported a gate‐tunable p–n diode composed of a BP/monolayer MoS_2_ heterostructure for excellent broad‐band photodetection and solar energy harvesting (**Figure** **19**).^254^ Upon illumination, these ultrathin diodes exhibited the highest photodetection responsivity of 418 mA W^−1^ at 633 nm and photovoltaic energy conversion with an external quantum efficiency of 0.3%. In addition, Xu's group demonstrated a BP/MoS_2_ hybrid‐based photodetector over visible to near‐infrared region.^255^ The electrical characteristics were electrically tuned by a gate voltage to achieve a wide range of current‐rectification performance with a forward‐to‐reverse bias current ratio at more than 10^3^. Meanwhile, the hybrid photodetector exhibited a microsecond response with the photoresponsivities of ≈22.3 and 0.1534 A W^−1^ at 532 nm and 1.55 µm, respectively.

Recently, Liu's group reported the photothermal anisotropy of BP‐ReSe_2_ heterostructures as a function of stacking angles, resulting from their optical anisotropy by the controlled integration of different 2D materials.^256^ In Lv's work,^257^ thin BP sheets were mechanically exfoliated onto a polydimethylsiloxane film and then transferred onto a HfO_2_/Si substrate with the earlier prepared electrical pads. Further, few‐layer SnSeS flakes were similarly exfoliated onto a polydimethylsiloxane film and then artificially stacked atop the BP sheet by using the optical microscope with assistance of an aligned transfer system, exhibiting different diode behaviors by adjusting BP channel length and back‐gate modulation.

#### 5.1.5 TMD Nanosheets with TMO Nanosheets and Others

**Figure 20:**
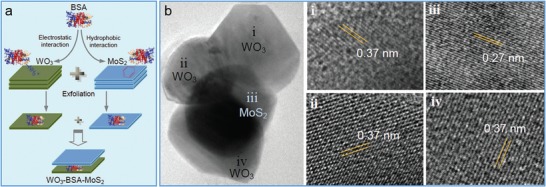
Surface hybridization of WO_3_ and MoS_2_ nanosheets into WO_3_/MoS_2_ hybrid by simultaneously exfoliating them in the presence of BSA. a) Schematic formation for WO_3_/MoS_2_ nanosheet sandwiched with BSA. b) Low‐resolution TEM image of a WO_3_/MoS_2_ hybrid prepared at the weight ratio of WO_3_/MoS_2_ = 4:1, accompanied with the high‐resolution TEM images on its individual nanosheets. Reproduced with permission.^42^ Copyright 2017, Wiley‐VCH.

With the same compositions of transition metals, the hybridization of TMD and TMO in 2D forms has been conveniently realized by partly oxidizing TMD nanosheet into TMD/TMO hybrids. Based on this strategy, TMO was successfully incorporated on TMD nanosheets for optimizing the gas sensing behavior by their synergistic effects.^258^ Similarly, Zhang's group prepared MoS_2_–MoO_3_ hybrids via a heat‐assisted partial oxidation of chemically exfoliated MoS_2_ sheets in air and subsequent thermal annealing‐driven crystallization.^259^ The resulting MoS_2_–MoO_3_ hybrids exhibited p‐type conductivity and further hybridized with n‐type SiC substrate to fabricate a p–n junction heterostructure for making light‐emitting diodes. In Bessonov's work, a novel memristive device was fabricated by sandwiching a MoO*_x_*/MoS_2_ or WO*_x_*/WS_2_ (*x* < 3) heterostructure between two printed silver electrodes, exhibiting an unprecedentedly large and controlled electrical resistance range from 10^2^ to 10^8^ Ω as well as low programming voltages at 0.1–0.2 V.^260^ In our recent work, a facile BSA‐mediated strategy was developed to successfully exfoliated WO_3_ and MoS_2_ nanosheets in BSA solution and further hybridize them to sandwiched WO_3_‐BSA‐MoS_2_ nanostructures via electrostatic and hydrophobic interactions, respectively (**Figure** **20**a).^42^ Experimentally, the simultaneous exfoliation of WO_3_ and MoS_2_ nanosheets under the assistance of BSA led to the effective hybridization of WO_3_ and MoS_2_ nanosheets (Figure 20b), which extended photoresponsivity of 2D nanomaterials to a wider spectral range from near‐infrared, visible to ultraviolet region.

### 5.2 Hybridization of Graphene with Others

Besides TMD nanosheets, graphene has also been used for hybridizing with other 2D nanosheets (e.g., BN, C_3_N_4_, and TMO) to achieve their synergistic effects in various enhanced applications. This section describes the hybridization of graphene with other 2D materials excluding TMDs as summarized earlier in last section.

#### 5.2.1 BN Nanosheets

Recently, graphene/BN heterostructures in the form of either in‐plane or inter‐plane hybrids have attracted numerous attention due to their outstanding performance in electronic devices where BN nanosheets are usually served as superb dielectric substrates or separation layers.^261^ In the van der Waals heterostructures, the hyperbolic plasmon–phonon polaritons can be effectively modulated by direct nanoinfrared imaging, originated from the hybridization of surface plasmon polaritons in graphene with hyperbolic phonon polaritons in BN.^262^ As a result, the graphene/BN hybrid was categorized as an electromagnetic metamaterial and its resulting behaviors were not presented in its constituent elements alone.

#### 5.2.2 C_3_N_4_ Nanosheets

In Qiao's work, 2D porous g‐C_3_N_4_ layers were integrated with N‐doped graphene via a simple vacuum filtration method.^263^ The resulting flexible 3D hybrid film was used as an electrocatalyst to display an unbeatable HER behavior with a very positive onset‐potential close to that of commercial Pt materials, including high exchange current density of 0.43 mA cm^−2^ and good durability with no loss of activity >5000 cycles, associated with excellent structural properties such as abundant active sites, hierarchical porous structure, synergistic coupling in hybrids, and nitrogen doping modification of graphene functionality. In Chen's work, C_3_N_4_/RGO hybrids were produced via a layer‐by‐layer assembly method to fabricate a paper‐sensing chip for selective detection of NO_2_ and SO_2_ gas by using “light on” and “light off” fashions, respectively.^264^ Without light irradiation, the sensor showed a p‐type semiconducting function to detect NO_2_ as low as 100 ppb without response toward SO_2_. Under UV light irradiation, the sensor exhibited n‐type semiconducting function instead to detect SO_2_ with a detection limit of 2 ppm. Further, semiconductor nanocrystals were introduced into the graphene/C_3_N_4_ hybrids to form ternary CdS/RGO/C_3_N_4_ hybrid photocatalyst for hydrogen generation and atrazine degradation through Z‐scheme electron transport, which originated from the coupling of two visible light‐active CdS/RGO nanohybrids and exfoliated C_3_N_4_ nanosheets.^265^ In another work, CdS nanorods were facilely sandwiched between g‐C_3_N_4_ and RGO nanosheets via a wet‐chemical approach.^266^ Owing to the matched band structure and close interfacial contact, a remarkable synergic effect was achieved for accelerating the separation and transfer of photogenerated charge carriers and contributing to an impressive photocatalytic performance and photostability under visible light irradiation. The dual interfaced nanocomposite showed hydrogen production rate of ≈4800 mmol h^−1^ g^−1^, which was 44, 11, and 2.5 times higher than those for single interfaced C_3_N_4_ nanosheets, C_3_N_4_/RGO and C_3_N_4_/CdS heterostructures, respectively.

#### 5.2.3 Other Types of Nanosheets

In Zhang's work, RGO‐wrapped MoO_3_ composite was facilely and conveniently fabricated by simply mixing Mo‐MOFs with GO nanosheets followed by an annealing process.^267^ The resulting porous RGO/MoO_3_ hybrids were utilized as a novel electrode material in all‐solid‐state, flexible and symmetric supercapacitors. Sasaki's group synthesized an alternately stacked MnO_2_/graphene superlattice‐like structure by a solution‐based assembly.^268^ All negatively charged MnO_2_ nanosheets were stabilized between positively charged poly‐(diallyldimethylammonium chloride)‐modified graphene monolayers, rather than the random stacking. After utilized in Li and Na storage, the superlattice‐like materials exhibited large specific capacities, high‐rate capacities and excellent cycling stabilities.

Recently, BP/graphene hybrid was also prepared via sandwiching BP sheets between graphene layers as a high‐capacity anode in Na‐ion batteries with various excellent performances, for example a specific capacity of 2440 mA h g^−1^ was obtained at a current density of 0.05 A g^−1^ and 83% capacity was still retained after operating between 0 and 1.5 V for 100 cycles.^269^ The behavior with large capacity was attributed to the intercalation of Na^+^ ions along the *x*‐axis of BP sheets and the subsequent creation of Na_3_P. Meanwhile, the hybridized graphene worked as a mechanical backbone and an electrical highway, providing a suitable elastic buffer space to accommodate the anisotropic expansion of BP sheets along the *y* and *z* axial directions for cycling operation with high stability. In Yu's work, GO/Bi_2_WO_6_ composite was hydrothermally synthesized and then chemically reduced to RGO/Bi_2_WO_6_ composite with ethyl glycol followed by photochemical loading of Ag nanoparticles under xenon lamp irradiation, and the resulting Ag/RGO/Bi_2_WO_6_ composite exhibited an enhanced visible light photocatalytic activity.^270^

### 5.3 Hybridization of BP Nanosheets with Others

Here we summarize more hybridizations of BP with other 2D nanomaterials including BN and C_3_N_4_, beyond TMDs and graphene described above. Typically, the hybridization of BN layers on BP sheets not only solved the instability of BP sheets, but also provided more novel properties. Constantinescu et al. simulated large and rotated BN/BP interfaces with linear‐scaling DFT and suggested that the main electronic features of BP nanosheets were preserved after interfaced between BN layers while BN spacers can counteract the bandgap reduction in stacked BP layers.^271^ Under the guidance of this supposition, a model for a tunneling FET was proposed by using BN‐spaced BP bilayers, which sustained both low‐power and fast‐switching performances, as those encountered in TMD‐based FETs. In another work, Vitiello's group sandwiched a BP sheet between multilayered BN nanosheets to fabricate BN/BP/BN heterostructured terahertz photodetectors with a high optical response and an extremely good time‐dependent electrical stability.^272^ In addition, heterostructured photocatalysts composed of BP and C_3_N_4_ were facilely produced by a one‐step liquid exfoliation approach.^273^ The combination of BP with C_3_N_4_ strengthened the visible light harvesting ability and facilitated the charge separation in photocatalytic process, rendering the promoted activity of photoinduced molecular oxygen such as superoxide radicals evolution and hydrogen peroxide production.

## 6 Conclusions and Outlook

In summary, we systematically overview a variety of hybridization methodologies for functionalizing diversified 2D nanomaterials to fabricate various 2D hybrids with new/improved properties and enhanced applications. Among them, the functionalized hybridization on TMD nanosheets are presented in detail through either physical or chemical doping, linkage, adsorption, and hybridization with other functional species (e.g., atoms, ions, molecules, polymers, and 0D, 1D, and 2D nanostructures) into or onto nanosheets, which either tunes the electronic, optical, and magnetic properties of TMD nanosheets, or manipulates their chemical solubility/dispersity in solutions and compatibility to other functional moieties and biological systems. At the same time, representative hybrids of other 2D nanomaterials are also introduced selectively based on their specific surface structures and requirements: hybridized graphene with carbon materials, metal and metal oxides, functionalized C_3_N_4_ nanosheets with protons, molecules and nanomaterials, and other hybrids of BP and TMO nanosheets with respective improved stabilization/enhanced applications and high solar‐harvesting capability. As an emerging field in 2D hybrid materials, the vertical stacking and in‐plane concatenating heterostructures of different types of nanosheets are summarized to exhibit their exciting physical phenomena upon the redistribution of charge and utilization for fabricating advanced electronic and optoelectronic devices.

Although these important developments definitely show the synergistic hybridization with other species is very effective to functionalize 2D materials and alter their physical and chemical properties, the current researches are still at an infancy stage so far with several identified challenges and opportunities in future exploitation of hybridized nanomaterials for extending their applications. Clearly, most of reported hybrids involve the use of nanosheets in few layers rather than monolayer, which restrict to discover some unique properties and features of composites that only lie in the form of monolayer nanosheets. Until now, it remains as a great challenge to produce monolayer nanosheets at high yield and large scale for most of layered materials. To retard another notable issue, there is a great demand to increase/improve controllability or reduce difficulties in hybridization of 2D materials for making significant breakthroughs such as unprecedented performance and unknown functional composites. For instance, the reported strategies cannot realize uniform distribution of additive species and adjust their arranging density and size on nanosheets. Also, weak noncovalent binding rather than strong covalent‐linkage are widely employed for achieving less synergistic hybridization from two or multiple components. Further, it will be significant to explore in control of inter distance between external species and nanosheets for creating novel hybrids that have not reported in literature. It is advisable to make more efforts for inserting intelligent molecules (e.g., stimuli responsive materials) or functional nanostructures (e.g., different size) between two adjacent 2D nanosheets via covalent bonding, similar to other combination systems.

It is known that the oriented growth or arrangement of anisotropic nanostructures (e.g., nanorods and nanobelts) on 2D nanomaterials along a special direction is very interesting but not achieved yet until now, and it will potentially arouse more novel properties via specified synergistic effects. Very recently, a sensational work published by Jarillo‐Herrero's group found an unconventional superconductivity in the twisted bilayer graphene with a twist angle of ≈1.1° (i.e., a magic angle in graphene superlattice),^274^, ^275^ which exhibit flat bands near zero Fermi energy in its electronic band structure and lead to correlated insulating states at half‐filling and zero‐resistance states at a critical temperature of up to 1.7 K. Inspired by the pioneering works, more works are rapidly performed and developed into an emerging field of twistronics, which open abundant opportunities to exploit the outstanding properties of 2D heterostructures hybridized in different formats by using the same or different types of nanosheets. To this end, the state of the art techniques are very pivotal for manipulating the accurate location/position of 2D nanomaterials and triggering greatly improved performance in diverse applications.

Of noted, the current works mainly involve binary hybrids whereas ternary or even quaternary hybrids are rarely reported for significantly surpassing the properties and functionalities of the binary counterparts. The other pursuing goals in exploitation of 2D hybrid systems are driven by their targeted applications through the rational choice and hybridization of 2D nanomaterials with other functional species in a predestinate manner. With the rapid progress in developing innovative nanosynthetic techniques and advanced surface‐modified methods, the facile but general syntheses of such hybrids will likely come true in the near future, rendering the hybridized 2D nanomaterials even more exciting and promising for vast applications.

## Conflict of Interest

The authors declare no conflict of interest.
